# The Tumor Suppressive Role of eIF3f and Its Function in Translation Inhibition and rRNA Degradation

**DOI:** 10.1371/journal.pone.0034194

**Published:** 2012-03-23

**Authors:** Fushi Wen, Renyuan Zhou, Alex Shen, Andrew Choi, Diana Uribe, Jiaqi Shi

**Affiliations:** 1 Department of Pathology, Department of Surgery, The University of Arizona Cancer Center, University of Arizona, Tucson, Arizona, United States of America; 2 Department of Urology, Fifth People's Hospital of Shanghai, Shanghai, People's Republic of China; The Chinese University of Hong Kong, Hong Kong

## Abstract

Deregulated translation plays an important role in human cancer. We previously reported decreased eukaryotic initiation factor 3 subunit f (eIF3f) expression in pancreatic cancer. Whether decreased eIF3f expression can transform normal epithelial cells is not known. In our current study, we found evidence that stable knockdown of eIF3f in normal human pancreatic ductal epithelial cells increased cell size, nuclear pleomorphism, cytokinesis defects, cell proliferation, clonogenicity, apoptotic resistance, migration, and formation of 3-dimensional irregular masses. Our findings support the tumor suppressive role of eIF3f in pancreatic cancer. Mechanistically, we found that eIF3f inhibited both cap-dependent and cap-independent translation. An increase in the ribosomal RNA (rRNA) level was suggested to promote the generation of cancer. The regulatory mechanism of rRNA degradation in mammals is not well understood. We demonstrated here that eIF3f promotes rRNA degradation through direct interaction with heterogeneous nuclear ribonucleoprotein (hnRNP) K. We showed that hnRNP K is required for maintaining rRNA stability: under stress conditions, eIF3f dissociates hnRNP K from rRNA, thereby preventing it from protecting rRNA from degradation. We also demonstrated that rRNA degradation occurred in non-P body, non-stress granule cytoplasmic foci that contain eIF3f. Our findings established a new mechanism of rRNA decay regulation mediated by hnRNP K/eIF3f and suggest that the tumor suppressive function of eIF3f may link to impaired rRNA degradation and translation.

## Introduction

Deregulated translation plays an important role in human cancer [1]. The translation process can be divided into 4 phases: initiation, elongation, termination, and ribosome recycling [2]. Translation is mostly regulated at the initiation phase. Eukaryotic initiation factor (eIF) 3 plays a central role in translation initiation.

Mammalian eIF3, the largest of the initiation factors, exists as a protein complex with at least 13 nonidentical subunits (eIF3a-m) [3]. The functions of the individual subunits have not yet been fully defined in mammals. Altering the expression level or the function of eIF3 may influence the synthesis of some proteins and consequently cause abnormal cell growth and malignant transformation. Seven eIF3 subunits have been implicated in human cancer [4], [5], [6]. Recent studies indicate that individual overexpression of 5 subunits of eIF3 promotes malignant transformation of NIH3T3 cells [7]. Therefore, deregulation of eIF3 subunits can contribute to tumorigenesis via induction of protein synthesis. However, how these eIF3 subunits contribute to tumorigenesis is still unclear.

The function of eIF3f, a non-core eIF3 subunit, is not well understood. Previously, we identified eIF3f as a protein involved in apoptotic signaling [8]. We demonstrated that eIF3f expression significantly decreased in many human cancers [6], [9], [10]. We also showed that restored eIF3f expression in tumor cells causes ribosomal RNA (rRNA) degradation, inhibits translation and cell proliferation, and induces apoptosis [6]. Those results represented the first demonstration that eIF3f contributes to tumorigenesis.

rRNA is an essential structural and catalytic component of ribosome. An increase in the rRNA level might promote the generation of cancer [11]. The homeostasis of the rRNA level must be maintained for normal cellular function and under stress conditions. Cells need to keep a balance between rRNA generation and degradation. The regulatory mechanism of rRNA degradation in mammals is not well understood. We previously showed that eIF3f might contribute to rRNA degradation [6]. However, the underlying molecular mechanism is not clear.

The heterogeneous nuclear ribonucleoprotein (hnRNP) K, an essential RNA and DNA binding protein, is a component of the hnRNP complex. We previously showed that hnRNP K is also involved in tumorigenesis [12], [13]. It is known that hnRNP K stabilizes RNA by binding to the 3′ UTR of the mRNA [14]. Yeast 3-hybrid screens and RNA pull-down assays indicated that hnRNP K binds to 18S and 25S rRNA in yeast [15]. However, whether hnRNP K regulates rRNA stability in humans is unknown.

In our current study, we tested the hypothesis that eIF3f coordinates with hnRNP K to regulate rRNA degradation and that decreased eIF3f expression contributes to tumorigenesis by deregulating translation and apoptosis. We demonstrated that eIF3f directly interacts with hnRNP K. Under stress conditions, eIF3f dissociates hnRNP K from rRNA, thereby preventing it from protecting rRNA from degradation. We showed that rRNA degradation occurs in non-P body, non-stress granule cytoplasmic foci. We also showed that silencing of eIF3f promotes both cap-dependent and cap-independent/internal ribosome entry site (IRES)-dependent translation and cytokinesis defects. Our findings establish the physiologic role of eIF3f in rRNA degradation and translation, and suggest that the tumor suppressive function of eIF3f may link to impaired rRNA degradation and translation.

## Materials and Methods

### Ethics Statement

The use of human pancreatic cancer tissues in this study was approved by the University of Arizona institutional review board. Archival formalin fixed paraffin embedded tissues stored in the Gastrointestinal Specialized Programs of Research Excellence (GI SPORE) Tissue Bank and Department of Pathology were used. Written informed consent from all participants involved in the study was obtained by the tissue bank.

### Cell culture and tissue specimens

We obtained BxPc3 and MiaPaCa-2 human pancreatic cancer cell lines from American Type Culture Collection (ATCC, Manassas, VA). The cells were cultured at 37°C with 5% CO_2_ in RPMI 1640 medium (Mediatech, Inc., Herndon, VA), supplemented with 10% fetal bovine serum (Omega Scientific, Inc, Tarzana, CA); 2.5 mg/ml glucose; 1% L-glutamine; and 1% penicillin/streptomycin (Invitrogen, Carlsbad, CA). Immortalized normal human pancreatic ductal epithelial (HPDE) cells and Kras^G12D^ HPDE cells were kindly provided by Dr. Ming-Sound Tsao, University of Toronto, Canada [16]. HPDE cells were cultured in keratinocyte serum-free medium supplemented with epidermal growth factor and bovine pituitary extract (Invitrogen). We also obtained primary human foreskin fibroblast (HFF)-1 cells from ATCC. The cells were cultured at 37°C with 5% CO_2_ in Dulbecco's Modified Eagle's Medium with 15% fetal bovine serum. All transfections were carried out using LipofectAMINE 2000 (Invitrogen) according to the manufacturer's instructions. Tissue specimens were obtained from the GI SPORE tissue bank in the Department of Pathology at the University of Arizona; our protocol was approved by the Human Subjects Committee of the University of Arizona.

### Construction of plasmids

We constructed pGEX-eIF3f and 4 pGEX-eIF3f deletion constructs as previously described [8]. By cloning full-length eIF3f into expression vector pcDNA3 (Invitrogen), using EcoRI and XhoI restriction sites, we constructed pcDNA3-eIF3f. pCMV-HA-eIF3f was constructed by cloning full-length eIF3f into expression vector pCMV-HA (Clontech Laboratories, Mountain View, CA), using EcoRI and XhoI restriction sites.

### Purification of recombinant protein and GST pull-down assay

Purification of recombinant protein and the glutathione S-transferase (GST) pull-down assay was performed as previously described by our group [8]. Briefly, ^35^S-labeled *in vitro* transcribed and translated hnRNP K was incubated with GST, with full length eIF3f, or with 4 deletion mutants of eIF3f. Then the bound hnRNP K was separated by SDS-PAGE and visualized by autoradiography. To examine the loading of the GST fusion proteins, the gel was also stained with coomassie blue.

### Immunofluorescence and molecular beacon analysis

Immunofluorescence analysis was performed as previously described by our group [8]. To trigger oxidative stress and induce P-body and stress granule formation, we used 0.5 mM sodium arsenite. In some cases, we stained nuclei with mounting medium with 4′,6-diamidino-2-phenylindole (DAPI) (Vector Laboratories, Burlingame, CA). Rabbit Rck antibody was kindly provided by Dr. Roy Parker. Rabbit polyclonal Dcp1a antibody was kindly provided by Dr. Jens Lykke-Andersen. eIF4G antibody was purchased from Santa Cruz Biotechnology (Santa Cruz, CA). Goat eIF3f antibody was generated by our group as previously described [8]. The specificity of the eIF3f antibody was well proved by our previous publications, collaboration and sharing with other investigators [6], [8], [17]. hnRNP K antibody was purchased from Sigma-Aldrich (St. Louis, MO). Secondary fluorescein isothiocyanate (FITC)-tagged anti-goat, Texas Red -tagged anti-rabbit or anti-mouse antibody was purchased from Jackson ImmunoResearch (West Grove, PA) and a 1∶100 dilution was used. For Rck and Dcp1a antibody, we used a 1∶200 dilution; for hnRNP K antibody, a 1∶100 dilution; and for eIF4G and eIF3f antibody, a 1∶50 dilution.

To detect endogenous rRNA in cells, we used molecular beacons that are complementary to 18S or 28S rRNA sequences. Molecular beacons are reporter oligo molecules that contain a fluorophore on one end and a quencher on the other end with a short stem-loop structure [18]. This prevents these molecules from generating fluorescence until they hybridize with their target RNA. Thus molecular beacon improves signal to noise ratio and specificity. We purchased Carboxyfluorescein (FAM)-tagged high-performance liquid chromatography (HPLC)-purified molecular beacons from, and designed by, Sigma-Aldrich: 28S rRNA: 5′CGCGATCAGCAGGATTACCATGGCAACGA TCGCG [BHQ1] 3′ and 18S rRNA: 5′ CGCGATCACCAACTAAGAACGGCCATGCAGATCG CG [BHQ1] 3′. Before we performed our immunofluorescence studies, cells were fixed and incubated with 200 nM of molecular beacon at 37°C for 1 hour as described [19]. FITC, Cy3, and Rhodamine Red or Texas-Red-tagged secondary antibody was used. P-body-positive cells were counted in a total of at least 300 cells.

### RIP-RT-PCR assay

We used a modified method described by Evans et al. [20]. Briefly, cells were incubated in 10 ml of medium containing 1% (V/V) formaldehyde for 10 minutes at room temperature. To quench the reaction, we added 0.25 M glycine. Then, we harvested the cells; resuspended the pellet in radioimmunoprecipitation assay (RIPA) buffer (50 mM Tris-Cl pH 7.5, 1% NP40, 0.5% sodium deoxycholate, 0.05% SDS, 1 mM EDTA, 150 mM NaCl); and sonicated the pellet to lyse the cells. Insoluble material was removed by centrifugation and the lysates were precleared by incubating them with protein G beads, mouse IgG, and yeast tRNA (100 ug/ml). Next, the samples were centrifuged and the proteins were immunoprecipitated from the supernatant overnight at 4°C by adding hnRNP K or mouse IgG control antibodies and protein G beads. Same volume of the sample was put aside as total input without immunoprecipitation. The beads were harvested and washed 6 times in RIPA buffer, additionally containing 0.5 M NaCl and 1 M urea. Finally, the beads were resuspended in 100 ul of 100 mM NaCl and 1% SDS to elute RNA. Then, the RNA from the elution and from the total input was extracted using phenol/chloroform/isopropanol (24∶25∶1). Total input and precipitated RNA was isolated and treated with DNase to eliminate DNA contamination. We either reverse-transcribed RNA or, for a negative control, did not; then, we performed polymerase chain reaction (PCR) or quantitative real-time PCR using indicated primers. Immunoprecipitated rRNA was normalized against total input rRNA.

### Bicistronic luciferase reporter assay

Bicistronic luciferase reporter constructs were kindly provided by Dr. Davide Ruggero (University of California, San Francisco). To determine the linear range of luciferase production in the transfection of the bicistronic luciferase constructs, we performed a time course study and a dose response study. A range between 25 and 100 ng plasmids was determined to be linear (data not shown). We used 70 nanograms of the individual construct to transfect into eIF3f-silenced or control HPDE cells in 24-well plates in triplicate. After 24 hours, cells were lysed in passive lysis buffer (Promega, Madison, WI); the luciferase activity was measured according to the manufacturer's instructions for a dual-luciferase assay (Promega).

### Actinomycin D chase analysis

We seeded cells at about 30% confluence in 6-well plates for 24 hours, and then treated them with actinomycin D (0.5 µg/ml) to inhibit de novo transcription as described [21]. At the indicated time point, cells were harvested. Total RNA was isolated and treated with DNase before real time RT-PCR analysis was performed.

### Quantitative real-time RT-PCR

Total RNA was extracted using RNeasy Mini Kit (QIAGEN, Valencia, CA) and treated with DNase. Real-time RT-PCR was performed using iQ SYBR® Green Supermix Reagents (Bio-Rad, Hercules, CA) and amplified in a 480 Lightcycler system according to the manufacturer's instructions (Roche, Basel, Switzerland). Melting curve was used to determine the specificity of the PCR products. We used these primers: 18S rRNA-forward 5′-CTGCCCTATCAACTTTCGATGGTAG-3′, reverse 5′-CCGTTTCTCAGGCTCCCTCTC-3′; and 28S rRNA-forward 5′-TGTCGGCTCTTCCTATCATTGT-3′, reverse 5′-ACCCAGCTCACGTTC CCT ATTA-3′ as described previously [22]. We have tested different reference genes, such as Glyceraldehyde 3-phosphate dehydrogenase (GAPDH), β-actin, and RPL32. We chose GAPDH as the reference gene because it is very stable and comparable between cell lines, and it maintains a high expression level (data not shown). eIF3f, hnRNP K and GAPDH primers were previously described [6], [23]. The conventional ΔΔCt method was used to calculate the fold changes of mRNA or rRNA levels and normalized to GAPDH mRNA. Average results from 3 independent experiments were shown as mean ± SD.

### Colony assay and soft agar assay

Colony assay was performed as previously described [6]. Briefly, 1,000 cells were seeded in 100-mm plates in triplicate and incubated for 2 weeks to allow colonies to form. Then the media were removed and the colonies were stained with methylene blue solution (50% methanol and 0.5% methylene blue) at room temperature for 5 minutes. The plates were rinsed with water and colony number was counted. For soft agar assay, cells were seeded at a density of 10,000 cells per well in 6-well cell culture plate in 2 ml 0.33% agar and cultured for 14 days at 37°C and 5% CO2. Colonies were then stained with 0.05% crystal violet overnight at 4°C by cover the soft agar with 1 ml of dye. Colonies were counted in the entire well.

### Cell survival, apoptosis, and cell cycle assay

Cell survival was measured by MTT (3-(4,5-Dimethylthiazol-2-yl)-2,5-diphenyltetrazolium bromide) assay as previously described [24]. For apoptosis assay, Caspase-Glo 3/7 Assay was used according to the manufacturer's instructions (Promega). Apoptosis was also measured by Annexin V staining and flow cytometry (FACScan, Becton Dickinson, San Jose, CA) or by proprion iodine staining and flow cytometry according to the manufacturer's instructions. Cell cycle was measured by flow cytometry after proprion iodine staining.

### Scratch assay

Cells were grown in 6-well plates until confluence was reached. Then, a gap was made by scratching the cells with a 200-ul pipette tip. Cell migration into the gap was observed and imaged over time.

### Cell migration assay

Cells were cultured in a Transwell culture system. Cell culture top chamber with 8.0 µm pore size filter (BD Labware, Le Pont De Claix, France) was inserted into a 24-well plate (bottom chamber). 500 µl keratinocyte medium supplemented with EGF containing 10% FBS was added to the bottom chamber. 10^5^ cells were seeded in 100 µl keratinocyte serum- free medium in the top chamber. Cells were culture for 48 hours before the medium in the top chamber was siphoned off and moved to the bottom chamber containing 4% paraformaldehyde to fix cells for 10 minutes. Top chamber was rinsed in PBS and inverted for staining. 50 µl of 5% crystal violet in 25% methanol was applied onto the bottom of the filter of the top chamber and cells were stained for 10 minutes. Excess crystal violet was washed off by plunging the top chamber into distilled water in a beaker several times. Finish washing in a second beaker till water is clear. Cells on top side of filter (cell that did not migrate) were removed using a moist cotton swab. The filter was then air dried. Cells were counted at 40× magnification in 5–7 fields under an inversion microscope.

### Crystal violet staining

Cells cultured on cover slips were fixed in 4% paraformaldehyde for 10 min. Cover slip was rinsed with PBS once and covered with 0.5% crystal violet (Sigma-Aldrich, St. Louis, MO) in 20% methanol for 10 min. Then the cover slip was rinsed with water 3 times. The stained cover slip was mounted on a regular glass slide with KPL mounting medium (Gaithersburg, MD). All steps were carried out at room temperature.

### 3D culture of HPDE cells

3D culture of HPDE cells was performed as previously described [25]. Briefly, we added 40 ul of Growth Factor Reduced Matrigel (BD Biosciences, San Jose, CA) to each well of an 8-well glass chamber slide and let it solidify at 37°C. Then, we added, on top of the Matrigel bed, 5,000 cells/well in 400-ul keratinocyte serum- free medium supplemented with EGF and bovine pituitary extract containing 2% Matrigel. We grew the cells in a 5% CO_2_ humidified incubator at 37°C. After 2 weeks, using laminin V antibody, we performed immunofluorescent analysis followed by confocal microscopy, as previously described [25].

### Knockdown of endogenous eIF3f and hnRNP K

Endogenous eIF3f was stably knocked down using 5 predesigned MISSION eIF3f shRNA lentiviral particles (NM_003754), according to the manufacturer's instructions (Sigma-Aldrich). Pre-designed hnRNP K siRNA (sense: 5′- ggaacaagcauuuaaaaga-3′, antisense: 5′-ucuuuuaaaugcuuguucc-3′) or negative control siRNA (50 nM) (Eurogentec, San Diego, CA) were transfected into the cells using Lipofectamine 2000 (Invitrogen), according to the manufacturer's instructions.

### Immunoprecipitation and Western blotting

Immunoprecipitation and Western blotting was performed as previously described [8]. The eIF3f antibody that we used was raised in a goat as previously described [8]. The CDK11 antibody was raised in a rabbit as previously described [8]. We purchased the hnRNP K monoclonal and α-tubulin antibody from Sigma-Aldrich and the cyclin B1 antibody from Santa Cruz Biotechnology.

### Immunohistochemistry analysis and Qdot staining

Immunohistochemistry analysis was performed as previously described [9]. We substituted regular secondary antibody with biotinylated secondary antibody and streptavidin-conjugated Qdot 655 (Tissue Acquisition and Cellular/Molecular Analysis Shared Service-TACMASS, University of Arizona Cancer Center). The nuclei were stained with DAPI in mounting medium.

### Coimmunoprecipitation and LC-MS-MS assay

Cell lysates were prepared from control and anti-Fas-treated A375 cells, then immunoprecipitated with eIF3f-specific antibody. Next, the immunocomplexes were subjected to 2-dimensional (2D) SDS-PAGE. Any protein spots on the anti-Fas-treated cell immunocomplex gel that appeared different from the control gel were cutted and submitted for digestion and proteomic analysis, using liquid chromatography-tandem mass spectrometry as previously described [26].

### Statistical analysis

All data are reported as mean ± standard deviation (SD). When appropriate, differences between 2 groups were compared by the *t* test. Differences were considered significant at *P*<0.05.

## Results

### Decreased eIF3f expression leads to malignant transformation of normal epithelial cells

**Figure 1:**
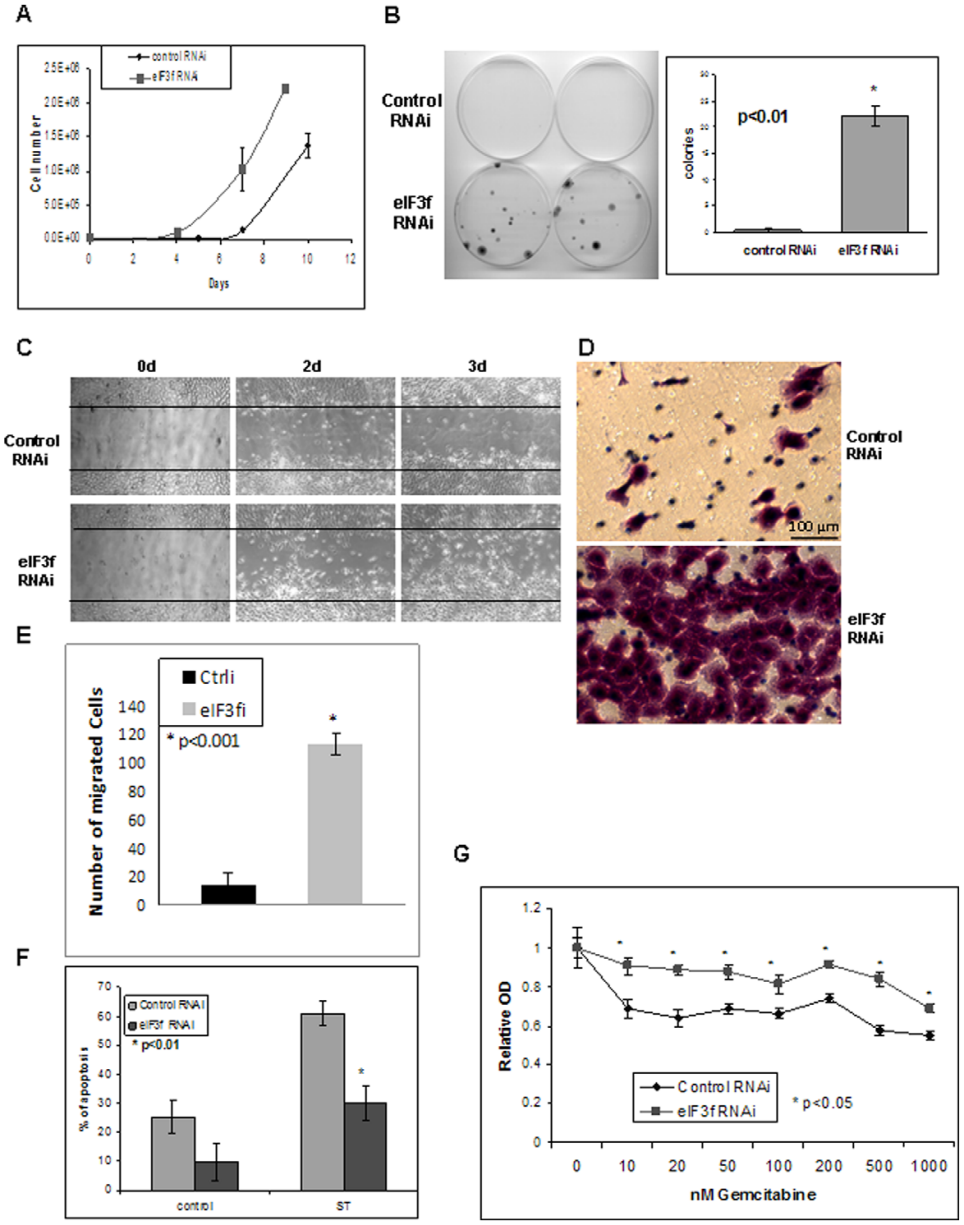
eIF3f-silencing in normal pancreatic epithelial cells led to malignant transformation. (A) In HPDE cells, eIF3f-silenced cells had a higher proliferation rate. Same number of eIF3f stable knockdown (clone #5) and control HPDE cells (5×10^4^ cells/plate) were seeded in triplicate on 100-mm plates and total cell numbers were counted every 2–3 days. (B) eIF3f-silenced cells formed more colonies. Same number (1000 cells) of eIF3f stable knockdown and control HPDE cells were seeded on 100 mm plates and colony formation was measured after 14 days. (C–E) eIF3f-silenced cells migrated faster than control cells. Confluent eIF3f RNAi or control RNAi HPDE cells were used for a scratch assay and pictures were taken at the indicated time as described in Materials and Methods (C). Same cells were also used for a migration assay using a Transwell culture system as described in Materials and Methods. A representative picture of the migrated cells stained with crystal violet on the filter was shown (D). Average number of migrated cells were counted and statistic difference between 2 cell lines was shown (E). (F) eIF3f-silenced cells were more resistant to apoptosis. eIF3f or control RNAi HPDE cells were treated with staurosporine (10 ng/ml) (ST) to trigger apoptosis. Apoptosis was measured after 24 h for ST using Annexin V staining and flow cytometry. Percentage of apoptotic cells out of total cells from 3 independent experiments was shown. (G) eIF3f-silenced cells were more resistant to gemcitabine treatment. eIF3f or control RNAi HPDE cells were treated with different concentrations of gemcitabine for 24 h before measuring the cell survival by MTT assay. The average ratio of gemcitabine/vehicle-treated cells OD from 3 independent experiments was plotted against gemcitabine concentrations.

**Figure 2:**
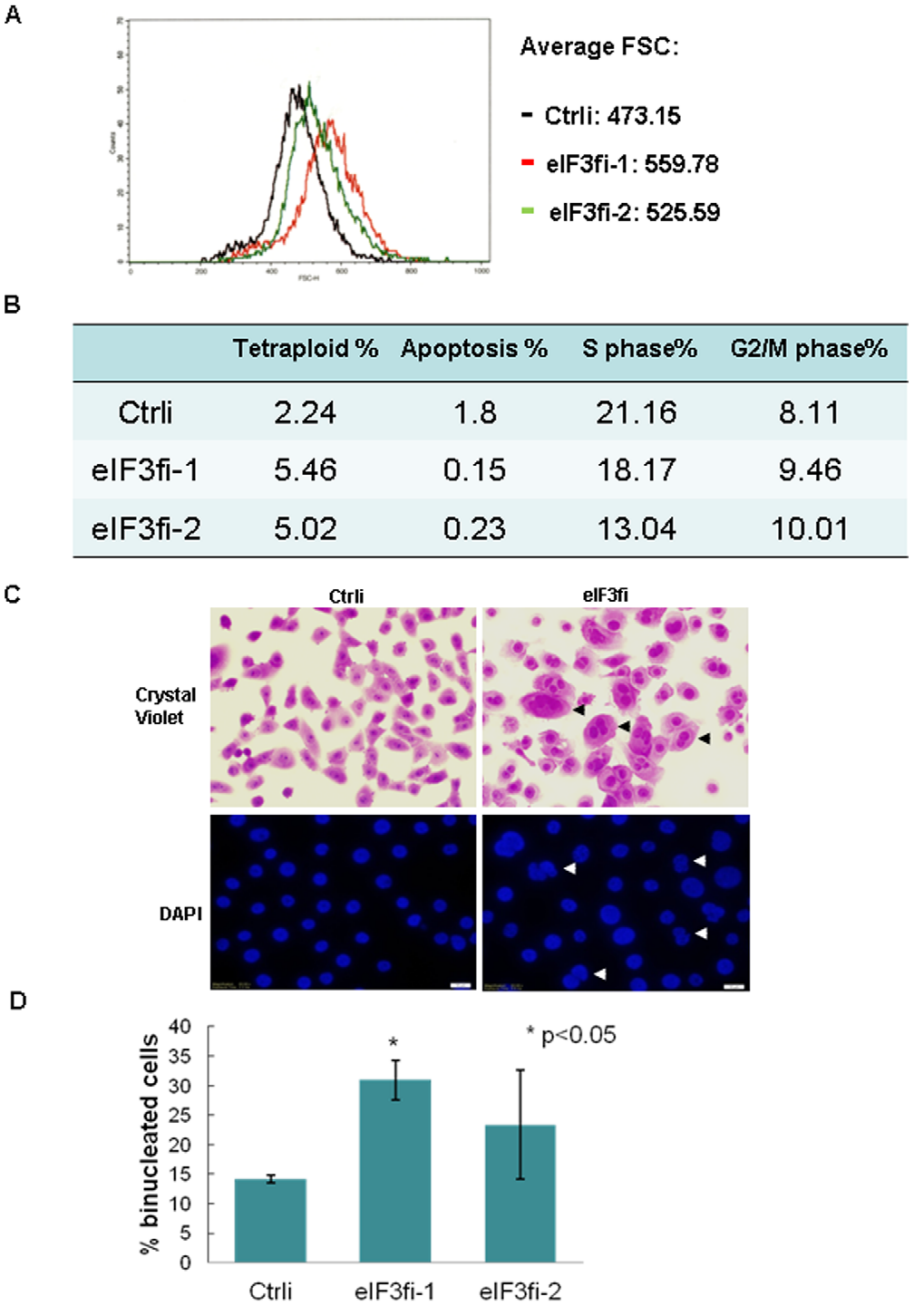
eIF3f-silencing led to increased cell size, nuclear pleomorphism, aneuploidy, and cell cycle abnormality. (A) eIF3f-silenced cells had an increased cell size. Two proliferating eIF3f-silenced clones (#4 and #5) and a control RNAi HPDE cell line were harvested and resuspended at 1×10^6^/ml in PBS solution. Cell sizes (indicated by forward scatter wave length-FSC) were measured by flow cytometry. (B) eIF3f-silenced cells had increased tetraploidy, increased number of G2/M phase cells, and decreased apoptosis. Cell cycle and apoptosis (indicated by sub-G1 peak) of 2 different eIF3f-silenced clones and a control RNAi HPDE cell line were measured by propion iodine staining and flow cytometry. Percentages of the indicated cells out of total cells were listed. (C) eIF3f-silencing produced increased binucleated/multinucleated cells (arrows). eIF3f-silenced cells and control HPDE cells were stained by crystal violet or DAPI. Representative images were shown. (D) Binucleated cells were counted in 2 different eIF3f-silenced clones and a control cell line. Average percentage of binucleated cells out of total cells from 3 independent experiments was shown.

Using quantum dot (Qdot)-labeled eIF3f antibody, we demonstrated that eIF3f was markedly decreased in pancreatic adenocarcinoma tissue, as compared with normal pancreatic ducts (Fig. S1A). Restoration of eIF3f expression in BxPC3 and MiaPaCa-2 pancreatic cancer cells induced apoptosis (Fig. S1B). However, it is unclear whether decreased eIF3f expression was the cause, rather than the consequence, of malignant transformation. To further investigate whether decreased eIF3f expression can transform normal epithelial cells, we stably knocked down endogenous eIF3f expression in the immortalized normal human pancreatic ductal epithelial (HPDE) cells, using 5 different eIF3f shRNA lentiviral transduction particles (Sigma-Aldrich) (Fig. S2A). HPDE cells are the only available immortalized human pancreatic ductal epithelial cell line that expresses normal level of eIF3f. They resembled the phenotype of normal cells rather than cancerous cells in vivo [27]. The proliferation of HPDE cells is anchorage-dependent, and they are not tumorigenic in SCID mice. We demonstrated that eIF3f mRNA was stably knocked down by 58% to 92% and eIF3f protein by 58% to 86% (Fig. S2A). For the following experiments, representative clone #5 or clone #4 and #5 were used unless otherwise stated. Stable knockdown of eIF3f in the HPDE cells increased cell proliferation, clonogenicity, apoptotic resistance, survival, resistance to a chemotherapy drug (gemcitabine), mesenchymal morphology, and migration (Fig. 1, Fig. S2B–D). Moreover, eIF3f-silenced cells also showed increased cell size, nuclear pleomorphism, aneuploidy, and cell cycle abnormality (Fig. 2). Note that increased cell size and aneuploidy caused by aberrant cytokinesis have been reported to be associated with aberrantly increased translation [28]. These features are supportive of malignant transformation in eIF3f-silenced HPDE cells.

**Figure 3:**
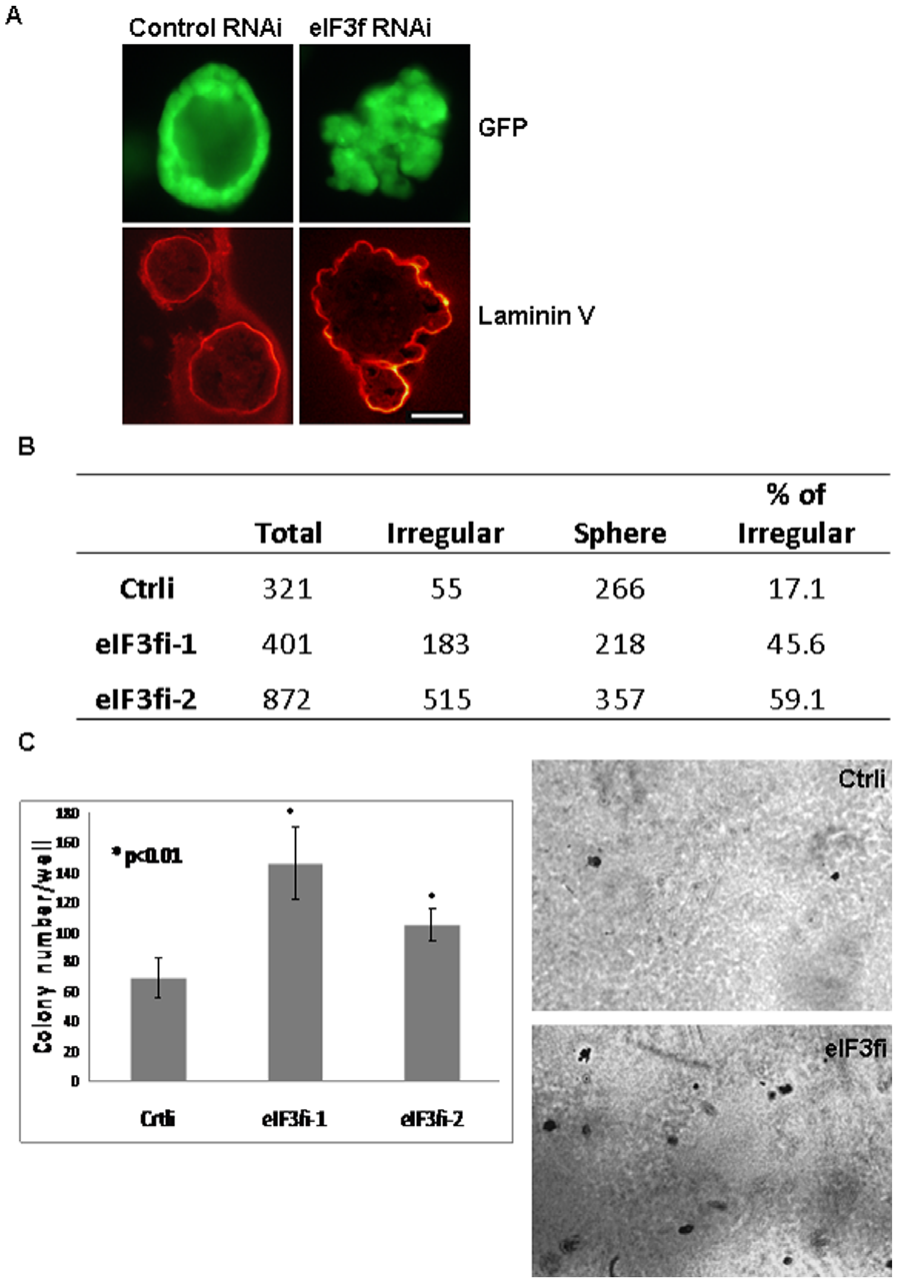
eIF3f-silenced HPDE cells showed malignant features in 3D culture and soft agar assay. (A) Control and eIF3f-silenced HPDE cells were stably transduced with a GFP-expressing lentivirus (pLKO-puro CMV-TurboGFP, Sigma-Aldrich) and positive cells were sorted by a cell sorter (FACSAria) (Fig. S3). These GFP-expressing cells were used in an ex vivo 3D- cell culture system, immunofluorescent and confocal microscopy analysis. Laminin V (indicating basement membrane) was labeled with laminin V antibody and secondary antibody conjugated with Texas Red. A representative picture of our 3D cell culture system is shown. Note the loss of normal architecture, of cellular polarity, and of smooth basement membrane in eIF3f-silenced HPDE cells. Bar: 50 µm. (B) We counted regular sphere and irregular structures of control cell lines and 2 eIF3f-silenced HPDE cell lines after 10 days of 3D culture. Note that eIF3f-silenced cells formed much more irregular structures than control cells. (C) Soft agar assay: control and eIF3f-silenced HPDE cells were seeded at a density of 10000 cells per well in 6-well plate in 2 ml 0.33% agar and cultured for 14 days. Colonies were stained with 0.05% crystal violet overnight at 4°C. Colonies in the entire well were counted. Representative images of colonies and histogram is shown.

Cells behave differently in 2-dimensional culture system versus 3-dimensional (3D) culture system. Malignant features of tumor tissue in vivo that distinguish from normal tissue include disrupted normal architecture and loss of polarity. To further confirm whether silencing of eIF3f transforms normal cells growing in a 3D environment mimicking in vivo situation, we used an ex-vivo 3D-cell culture system. We showed that eIF3f-silenced HPDE cells formed more (45–59% vs. 17% in control) irregular masses with abnormal architecture and polarity (recapitulating malignant tumors in vivo), while control cells developed into a single-layer epithelial hollow spheres (resembling normal pancreatic ductal structure in vivo) (Fig. 3A–B, Fig. S3). Furthermore, eIF3f-silenced HPDE cells proliferated in an anchorage independent manner by soft agar assay (Fig. 3C). These results support the hypothesis that decreased expression of eIF3f is an important cause of pancreatic cancer.

### eIF3f inhibits both cap-dependent and IRES-dependent translation

**Figure 4:**
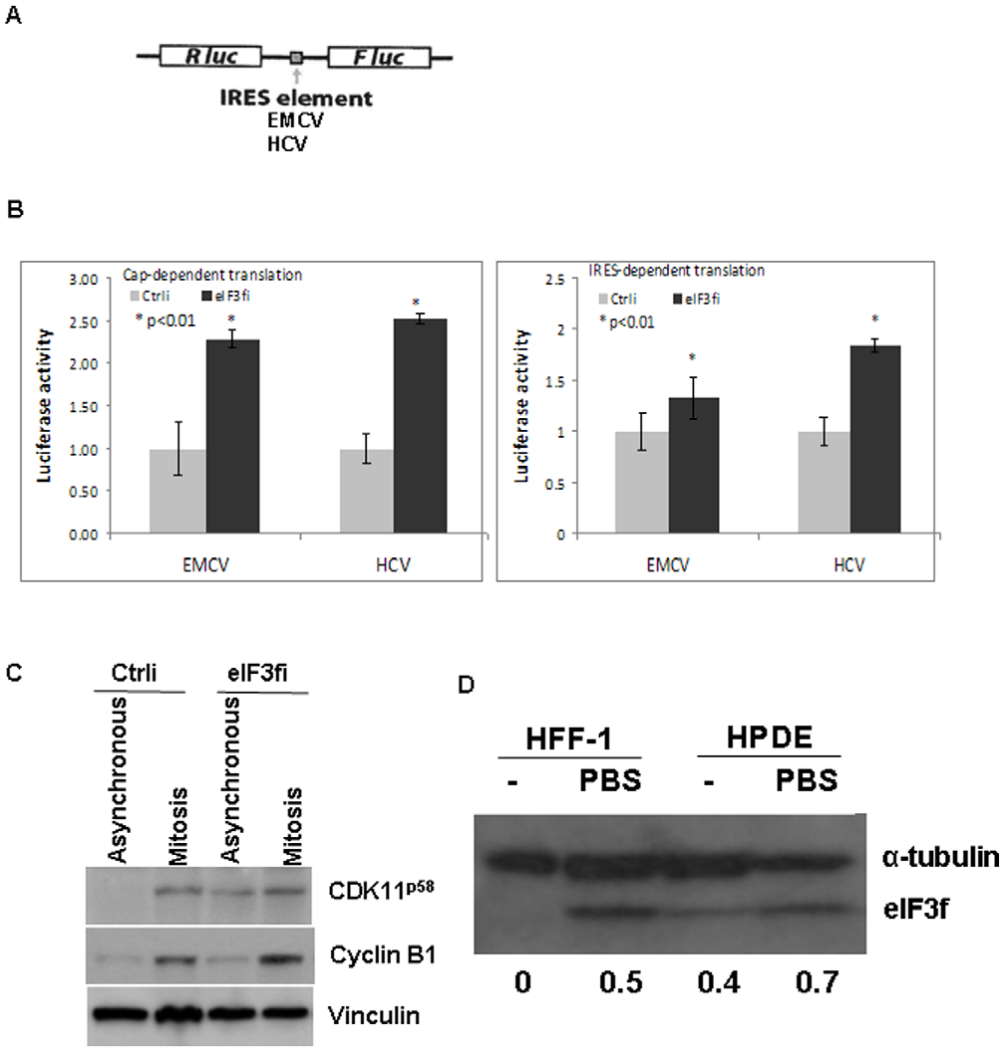
eIF3f inhibited both cap-dependent and IRES-dependent translation. (A) The map of the pRF bicistronic luciferase reporter (Renilla luciferase [Rluc], Firefly luciferase [Fluc]) constructs contained 1 of the 2 IRES elements of EMCV or HCV. (B) Same number of control and eIF3f-silenced HPDE cells were transfected with the same amount of 1 of the pRF bicistronic luciferase constructs. Dual luciferase assay for both firefly and renilla luciferase activity was performed 24 hours after transfection according to the manufacturer's instructions (Promega). Relative luciferase activity compared to control RNAi cells from 3 independent experiments was shown. (C) Control and eIF3f-silenced HPDE cells were either asynchronous or treated with nocodazole (40 ng/ml) for 16 h to block the cells in G2/M phase. Cells were harvested and Western blot was performed using CDK11, cyclin B1 and vinculin antibodies. Note that CDK11^p58^ expression was present only in mitotically synchronized control cells, but was also present in asynchronous eIF3f-silenced cells because of increased IRES activity in its 5′UTR. Cyclin B1 expression confirmed the G2/M phase. Vinculin was used as loading control. (D) Starvation led to increased eIF3f protein level. HFF-1 and HPDE cells either were cultured in regular medium or were starved for 6 hours in PBS before harvest. Western blot is performed to determine eIF3f protein level. Densitometric analysis of the eIF3f bands normalized to corresponding α-tubulin is shown at the bottom.

Translation initiation can be cap-dependent or cap-independent/IRES-dependent. Whether eIF3f inhibits cap-dependent or cap-independent translation is not known. To investigate the specific effect of eIF3f on translation, we measured both cap-dependent and cap-independent/IRES-dependent translation in eIF3f-silenced HPDE cells using a commonly used bicistronic luciferase reporter construct that contains both cap structure and one of the 2 viral IRES elements (EMCV or HCV) (kindly provided by Dr. Davide Ruggero, UC San Francisco) that require eIF3 (Fig. 4A) [28], [29]. Cap-dependent translation is indicated by Renilla luciferase activity (Rluc), and IRES-dependent translation is indicated by Firefly luciferase activity (Fluc). In our study, silencing of eIF3f increased both cap-dependent and IRES-dependent translation (up to 2.5-fold increase), indicating a suppressive role of eIF3f on both translation initiation mechanisms (Fig. 4B). The variation between the effect of eIF3f-silencing on EMCV and HCV IRES function can be explained by the different requirement of translation initiation factors: EMCV IRES requires eIF4G and eIF4A while HCV IRES does not.

An accurate IRES-dependent translation is required for mitotic progression [28]. For example, IRES-dependent translation of a CDK11 isoform (CDK11^p58^) facilitates accurate mitotic progression [30], [31]. In our study, we found that CDK11^p58^ expression was markedly increased in asynchronous eIF3f-silenced cells, as compared with control cells (Fig. 4C). This result was not due to an unequal mitotic rate, because cyclin B1, a mitotic enrichment indicator, was equally expressed at a low level in both asynchronous cells (Fig. 4C). Rather, eIF3f was required for the suppression of IRES-dependent translation, which, when impaired, results in the cytokinesis defect represented by increased binucleated and mitotic cells in eIF3f-silenced cells (Fig. 2). The function of eIF3f in inhibition of translation was also supported by our observation that starvation caused an increase in the eIF3f level, in both primary fibroblast cells and pancreatic epithelial cells (Fig. 4D). Starvation was known to lead to the inhibition of translation. In starved muscle myotubes, however, others have reported an association between suppressed translation leading to hypotrophy and a decreased eIF3f level, suggesting a tissue-specific role of eIF3f [6], [32], [33].

### eIF3f promotes rRNA degradation

**Figure 5:**
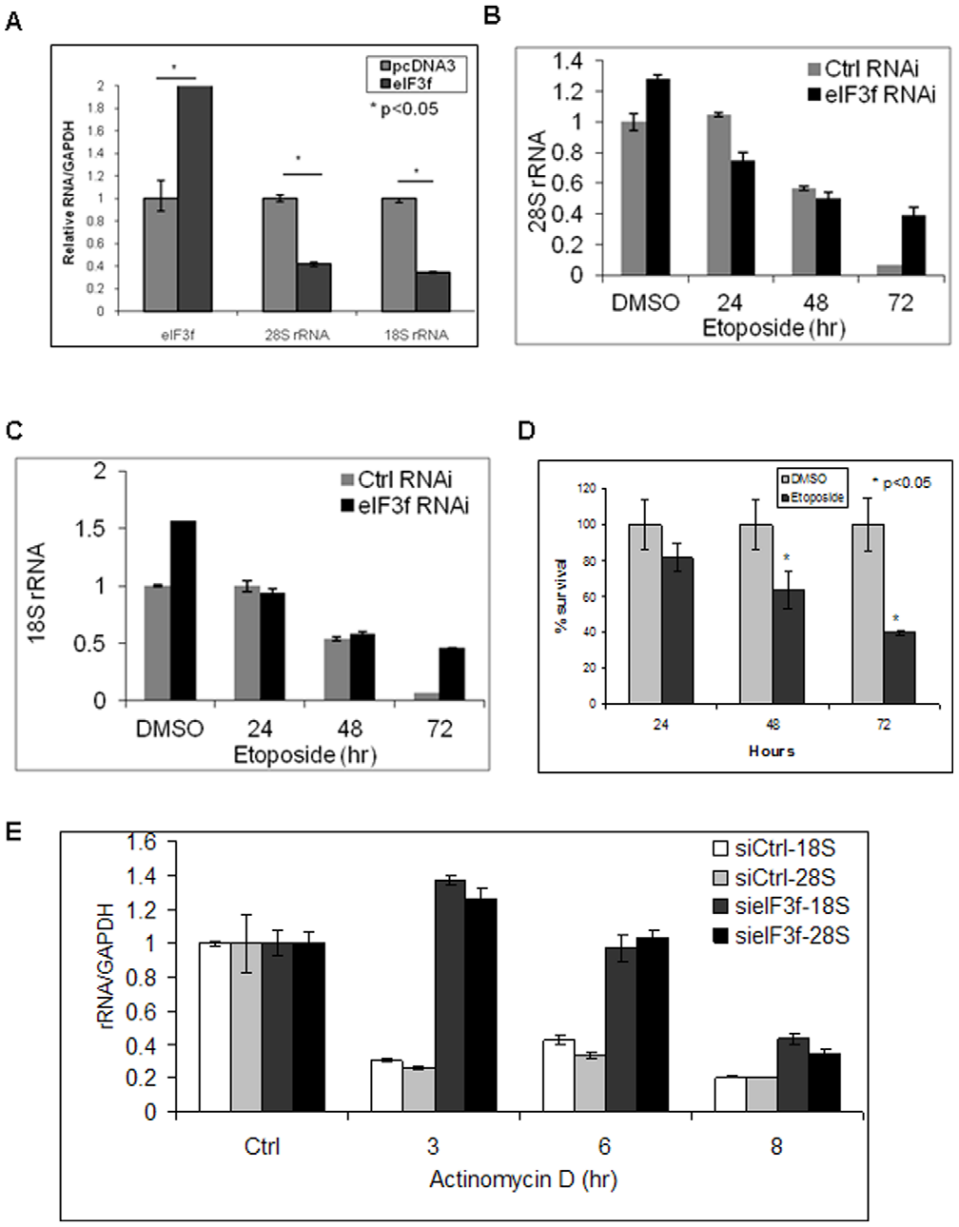
eIF3f induced rRNA degradation in pancreatic cells. (A) Restoration of eIF3f expression in pancreatic cancer cells decreased the rRNA level. MiaPaCa-2 cells were transfected with pcDNA3-eIF3f or pcDNA3. Relative fold changes of rRNA and eIF3f levels were quantified by real time RT-PCR and normalized to GAPDH mRNA. (B)(C) Silencing of eIF3f expression increased the rRNA level during apoptosis. Control and eIF3f-silenced HPDE cells were treated with DMSO vehicle control or etoposide (10 µM) for indicated time to induce apoptosis. Cells were harvested and total RNA was isolated using RNeasy Kit. Relative fold changes of 28S (B) and 18S (C) rRNA levels were quantified by real time RT-PCR after DNase treatment. (D) Time course of cell survival after etoposide treatment. HPDE cells were treated with DMSO or etoposide (10 µM) for indicated time. Cell survival was measured using MTT assay. Relative percentages of survived cells compared to DMSO-treated cells were shown. (E) Silencing of eIF3f expression attenuated rRNA degradation. Control and eIF3f-silenced HPDE cells were treated with actinomycin D (0.5 µg/ml) to block transcription for 3, 6 and 8 h before harvest. Total RNA was isolated, treated with DNase, and relative 28S and 18S rRNAs fold changes compared to control were quantified by real time RT-PCR and normalized to GAPDH mRNA.

We found that restoration of eIF3f expression in pancreatic cancer cells led to decreased rRNA, especially 28S rRNA (Fig. S4A). MiaPaCa-2 pancreatic cancer cell line has a decreased eIF3f expression [6], therefore was used in the following eIF3f-restoration experiments. Quantitative real time RT-PCR has been used to quantify rRNA or pre-rRNA level [22], [34], [35], [36]. In our study, quantification of the rRNA level by real time RT-PCR showed that 28S and 18S rRNA decreased up to 70% in eIF3f-restored MiaPaCa-2 pancreatic cancer cells (Fig. 5A, S4A). This rRNA decrease was not a result of apoptosis, because a significant rRNA decrease started as early as 4 hours after transfection, which occurred much earlier than the peak apoptosis (after 48 hours) (Fig. S4B, C). These results are consistent with our previous rRNA degradation observations in melanoma cells [6].

To further rule out the apoptosis effect on rRNA level and to assess if decreased eIF3f expression does the opposite, we used eIF3f-silenced HPDE cells to access rRNA level change during apoptosis. We found that the rRNA level was markedly higher in eIF3f-silenced cells than in control cells, by more than a 6-fold increase at 72 hours (Fig. 5B, C). Time course of the cell death was shown in Fig. 5D. To further prove that this higher level of rRNAs was due to decreased rRNA degradation rather than increased rRNA production, we used actinomycin D to block transcription and then followed the rRNA degradation over time course. Consistent with our previous observations, silencing of eIF3f markedly protected rRNA from degradation (up to 4-fold higher) (Fig. 5E). These results suggested that eIF3f may play an important role in regulating rRNA degradation.

### eIF3f directly interacts with hnRNP K

**Figure 6:**
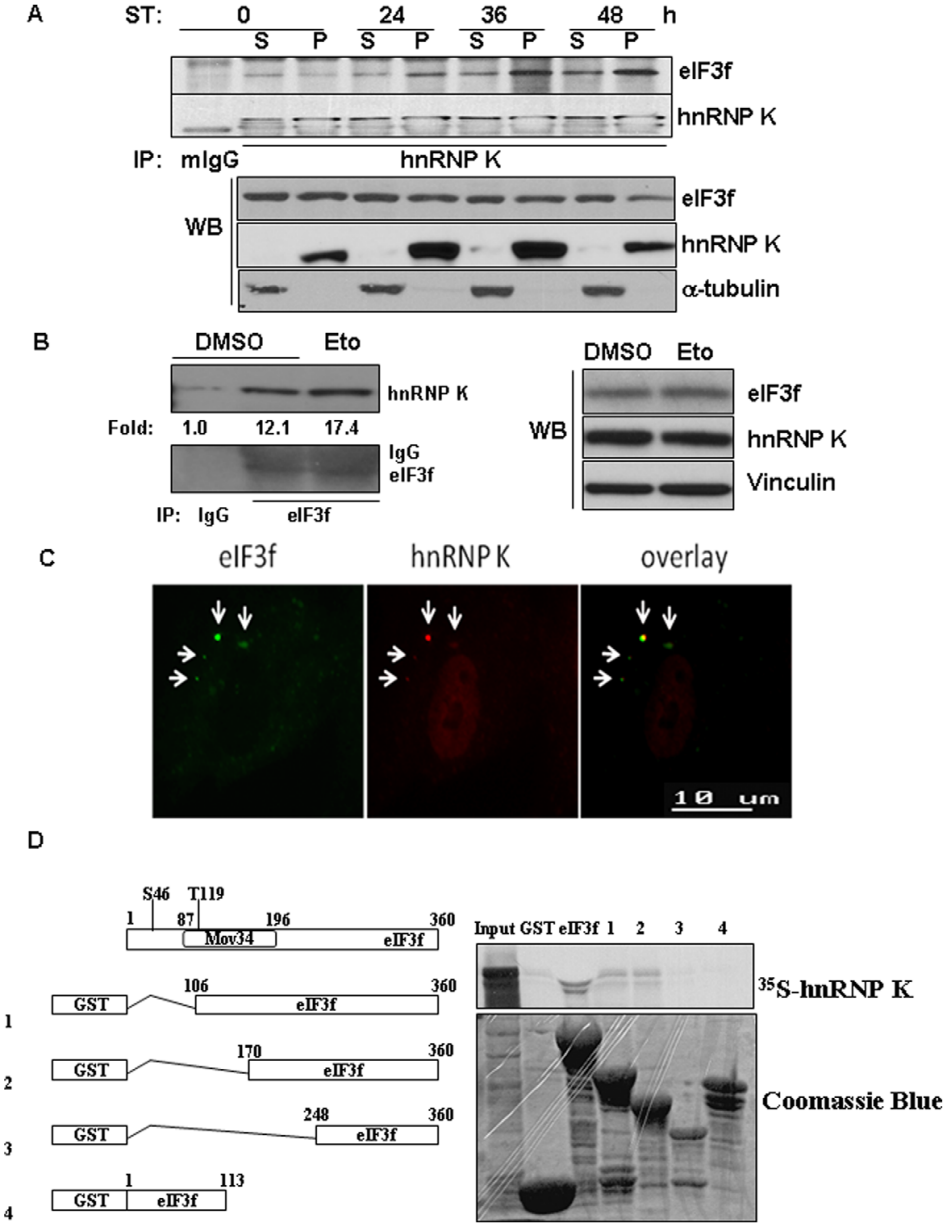
Increased endogenous interaction between eIF3f and hnRNP K under stress. (A) A375 cells were treated with staurosporine (ST) for the indicated time and cell fractionation was performed as previously described [17]. 500 µg of the lysates from the soluble (S) and pellet (P) fractions of the cells were immunoprecipitated with IgG or hnRNP K antibodies, followed by immunoblot with eIF3f and hnRNP K antibodies (top 2 panels). 50 µg of the same lysates were used for immunoblot with eIF3f, hnRNP K and α-tubulin antibodies without immunoprecipitation (bottom 3 panels). (B) HPDE cells were treated with DMSO or etoposide (Eto) (10 µM) for 24 hours. Cell lysates were immunoprecipitated with IgG or eIF3f antibodies, followed by immunoblot with eIF3f and hnRNP K antibodies (left 2 panels). The intensities of the bands were quantified using ImageJ software. The same cell lysates were used for immunoblot with eIF3f, hnRNP K and vinculin antibodies without immunoprecipitaiton (right 3 panels). (C) Immunofluorescent staining to eIF3f (FITC, green) and hnRNP K (Cy3, red) in HFF-1 fibroblasts treated with sodium arsenite (0.5 mM) for 30 minutes. (D) eIF3f directly interacted with hnRNP K. The left diagram shows full-length eIF3f and the location of the Mov34/JAB_MPN domain. Four GST-eIF3f truncation mutants were designed to localize the binding site of eIF3f with hnRNP K. GST pull-down assay was performed as described in the Materials and Methods.

To define the mechanism by which eIF3f degrades rRNA, we used a proteomic approach to identify proteins associated with eIF3f. We found that hnRNP K was one of the prominent proteins associated with eIF3f during apoptosis (data not shown). Three-hybrid screens and RNA pull-down assays suggested that hnRNP K binds to 18S and 25S rRNA in yeast [15]. However, whether hnRNP K regulates rRNA degradation in human is not known. Using co-immunoprecipitation analysis, we confirmed that endogenous eIF3f increased its interaction with hnRNP K during apoptosis in 2 different cell lines (Fig. 6A, B). We can also show that endogenous eIF3f was co-localized with hnRNP K in cytoplasmic foci in cells under stress (Fig. 6C, S5A). Furthermore, recombinant eIF3f directly interacted with in vitro transcribed and translated hnRNP K (Fig. 6D). Previously, we showed that the Mov34/JAB_MPN domain of eIF3f was involved in protein-protein interaction [8]; in our current study, we showed that this domain also mediated the interaction between eIF3f and hnRNP K (Fig. 6D). These results suggested a direct interaction between eIF3f and hnRNP K.

### hnRNP K binds to and stabilizes rRNA

**Figure 7:**
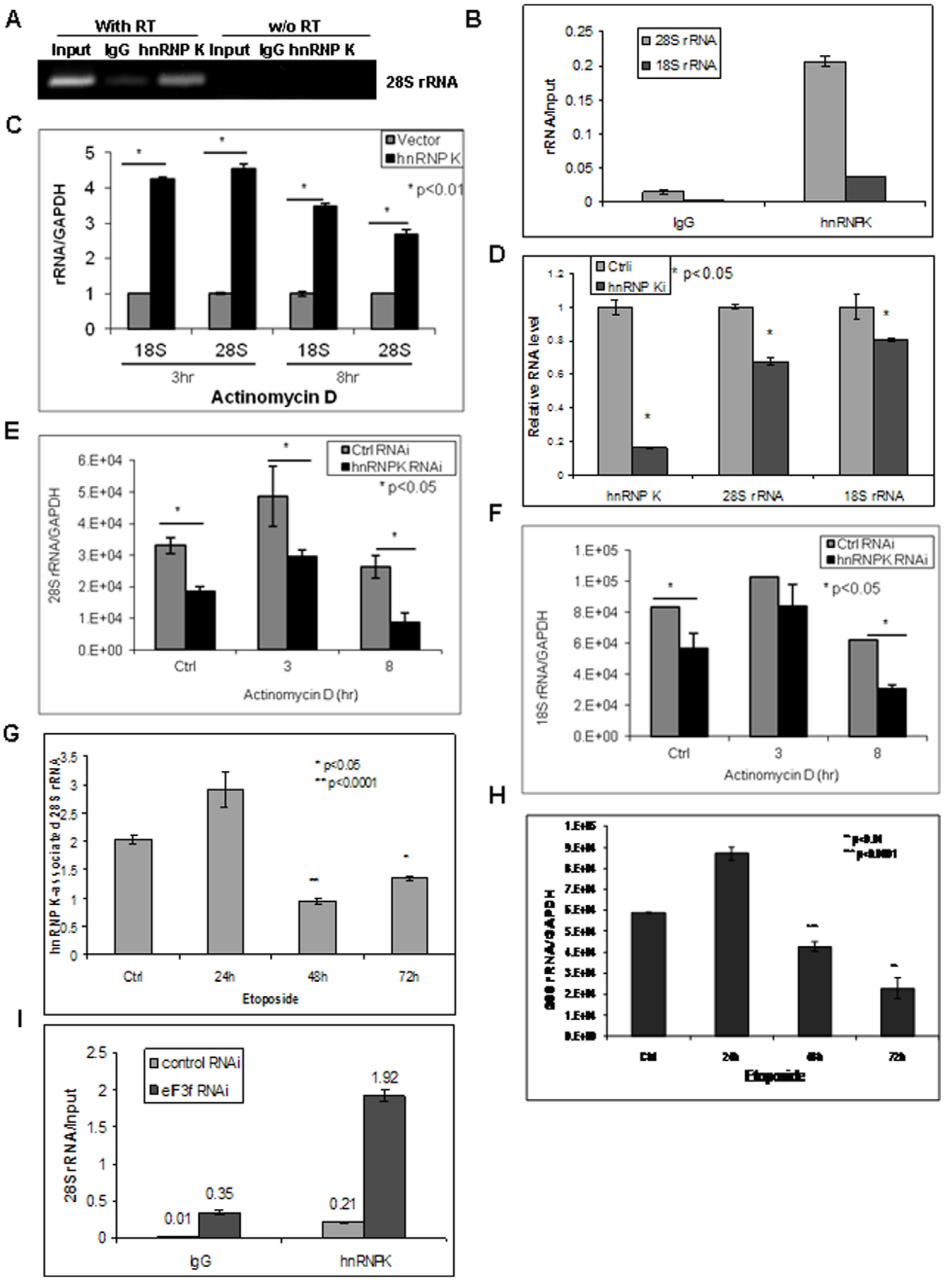
eIF3f regulated rRNA stability through hnRNP K. (A)(B) Endogenous hnRNP K bound to rRNA. RIP-RT-PCR analysis was performed in HPDE cells using IgG control or hnRNP K-specific antibodies as described in Materials and Methods. hnRNP K-binding rRNA was immunoprecipitated and eluted from the beads. Regular RT-PCR or PCR (without [w/o] RT as a negative control) (A) or quantitative real time RT-PCR (B) was used to identify the relative quantity of hnRNP K-binding rRNA. Input is the RT-PCR of the total RNA before immunoprecipitation as a positive control. IgG is the RT-PCR of the non-specific RNA that immunoprecipitated with the same species IgG antibody as a negative control. In (B), immunoprecipitated rRNA was normalized against total input rRNA for the same sample. (C) Ectopic expression of hnRNP K protected rRNA from degradation. HPDE cells were transfected with pcDNA4-hnRNP K or pcDNA4 control vector. Cells were treated with actinomycin D for 3 or 8 hours to block transcription 24 hours after transfection. Real time RT-PCR analysis was performed to quantify rRNA fold change normalized to GAPDH mRNA. (D) Silencing of hnRNP K decreased rRNA. Predesigned hnRNP K siRNA (50 nM, Eurogentec) was transfected into MiaPaCa-2 cells using Lipofectamine. rRNA and hnRNP K mRNA fold changes compared to control RNAi cells were assessed by real time RT-PCR after 48 hours. (E)(F) Increased rRNA degradation in hnRNP K-silenced cells. hnRNP K expression was knocked down in MiaPaCa-2 cells by siRNA as in (D). Actinomycin D was added to the cells 48 hours after transfection for 3 or 8 hours before harvest. Total RNA was isolated, DNase treated and real time RT-PCR analysis was performed to quantify 28S (E) and 18S (F) rRNA fold changes normalized to GAPDH mRNA. (G)(H) rRNA degradation was associated with decreased binding of hnRNP K with rRNA during apoptosis. HPDE cells were treated with etoposide (10 µM) for 24, 48 or 72 hours and hnRNP K-bound 28S rRNA (G) and total 28S rRNA (H) were assessed by RIP and/or real time RT-PCR assay. The fold changes of 28S rRNA were normalized to total input RNA (G) or GAPDH mRNA (H). (I) Silencing of eIF3f expression increased the binding of hnRNP K to rRNA. RIP-RT-PCR was performed in eIF3f-silenced or control HPDE cells using IgG or hnRNP K antibodies to assess the 28S rRNA level that binds to hnRNP K. The relative fold changes of hnRNP K-bound 28S rRNA was normalized to total input rRNA of the same sample.

To determine the function of hnRNP K in regulating rRNA degradation, we first performed RIP-RT-PCR analysis to assess whether hnRNP K binds to rRNA. We found that endogenous hnRNP K protein co-immunoprecipitated with rRNA in HPDE cells, indicating that hnRNP K bound to both 28S and 18S rRNA (Fig. 7A, B). To investigate whether hnRNP K protects rRNA from degradation, we ectopically expressed hnRNP K and observed rRNA degradation over time after blocking de novo transcription using actinomycin D. Increased hnRNP K expression dramatically protected rRNA from degradation (more than 4-fold) (Fig. 7C, S5B). In contrast, suppressed hnRNP K expression in MiaPaCa-2 pancreatic cancer cells significantly reduced the rRNA level and increased rRNA degradation (up to >60% decrease) (Fig. 7D–F, S5C). We have shown previously that hnRNP K was successfully knocked down in MiaPaCa-2 cells [23]. MiaPaCa-2 pancreatic cancer cells were chosen because we have reported that hnRNP K expression is increased in this cell line [23]. These results suggested, for the first time to our knowledge, that hnRNP K is essential and sufficient for the maintenance of rRNA stability in human epithelial cells.

To further investigate whether the binding of hnRNP K to rRNA correlates with rRNA degradation, we examined its binding to 28S rRNA using RIP-RT-PCR analysis during apoptosis. We saw a more than 50% decrease in hnRNP K-associated 28S rRNA at 48 and 72 hours after etoposide (Eto) treatment (Fig. 7G), which correlated with the time course of 28S rRNA degradation and cell death (Fig. 7H, S5D). These results suggested that dissociation of hnRNP K from rRNA may contribute to rRNA degradation during apoptosis.

### eIF3f interferes with the rRNA-protective function of hnRNP K

We found that hnRNP K protected rRNA from degradation, whereas eIF3f promoted rRNA degradation. We also showed that eIF3f interacted with hnRNP K during apoptosis. Thus, we hypothesize that eIF3f may promote rRNA degradation by interfering with the rRNA protective function of hnRNP K. To investigate this hypothesis, we compared the rRNA-binding capacity of hnRNP K in eIF3f-silenced HPDE cells and control cells. Indeed, silencing of eIF3f increased the binding of hnRNP K to 28S rRNA by more than 9-fold (Fig. 7I). This result supported our hypothesis that eIF3f interferes with the rRNA protective function of hnRNP K.

### Subcellular localization of eIF3f, hnRNP K, and rRNAs and their relationships with P bodies and stress granules

**Figure 8:**
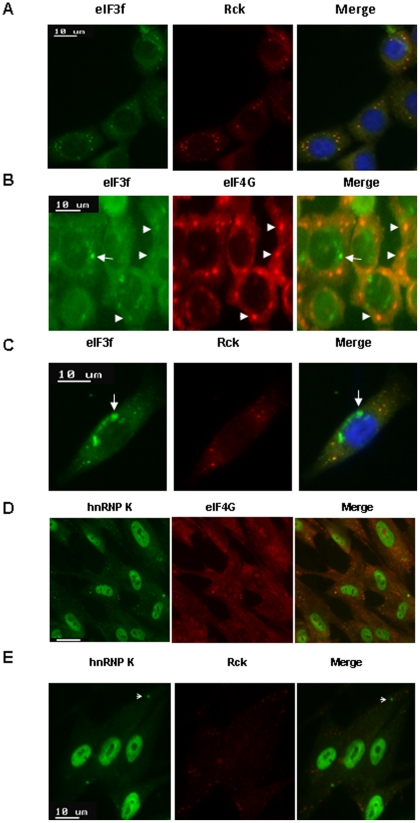
Localization of eIF3f/hnRNP K and their relationships with P body and stress granule. HPDE or HFF-1 cells were treated with sodium arsenite (0.5 mM) for 45 minutes to trigger P bodies and stress granules formation. (A)(B) Under stress, eIF3f was predominantly co-localized with both P bodies and stress granules. Immunofluorescent study was performed using eIF3f, P body markers (Rck, Dcp1a), or stress granule marker eIF4G antibodies and FITC (green) or Texas Red (red) -tagged fluorescent secondary antibodies in HPDE cells. Arrowhead indicated co-localization foci (yellow). Arrow indicated non-P body, non-stress granule foci. (C) eIF3f was also localized to non-P body, non-stress granule perinuclear foci (arrow) in HPDE cells. Same experiments as (A) were performed. (D)(E) hnRNP K was localized in stress granules and unknown cytoplasmic foci (arrow), but not in P bodies. Immunofluorescent study was performed using hnRNP K (mouse), Rck (rabbit), or eIF4G (goat) antibodies and FITC-tagged anti-mouse (green), Texas Red-tagged anti-rabbit (red) or Cy3-tagged anti-goat secondary antibodies (red) in HFF-1 cells. Bar: 10 µm.

Currently, it is not known where and how normal human rRNA is degraded during stress. Yeast nonfunctional rRNA decay is seen in P bodies and perinuclear foci [37]. Human translational stalled mRNAs were observed in P bodies (which generally contain the mRNA decay machinery), as well as in stress granules (which contain many translation initiation components, including eIF3). To determine the subcellular localization of eIF3f/hnRNP K and their relationships with P body and stress granule, we used arsenite stress to induce P body and stress granule formation. We found that eIF3f was colocalized with P-body markers (Rck, Dcp1a) in 91% of the cytoplasmic foci of arsenite-stressed HPDE cells [38] (Fig. 8A, S6A, S7A, S7E). We observed this phenomenon not only in normal HPDE cells, but also in BxPC3 pancreatic cancer cells (Fig. S6A). Such a surprising result challenges the current assumption that eIF3 only localizes to stress granules [38]. Our disparate result is probably due to the overlooked specific individual subunits of eIF3, such as eIF3f in our study. Not surprisingly, we found that eIF3f was also co-localized with stress granule marker eIF4G in 84% of the cytoplasmic foci (Fig. 8B, S7B). In addition, eIF3f accumulated to noticeable cytoplasmic and perinuclear foci that were not P bodies or stress granules (Fig. 8B, 8C, S6A–B, arrowheads). These results suggested that eIF3f is localized in both P bodies and stress granules, as well as in unknown cytoplasmic foci. Furthermore, eIF3f-silenced cells have up to 2.5-fold increase in P-body-bearing cells, suggesting that eIF3f may inhibit P body formation (Fig. S6C).

We also assessed the cellular localization of hnRNP K (in parallel with eIF3f) and its relationship with P bodies, stress granules, and perinuclear foci in HFF-1 and HPDE cells under stress. Other studies have shown that hnRNP present in both P bodies and stress granules [39]. However, our data suggested that hnRNP K was localized predominantly in stress granules (83% in HFF-1 cells and 99% in HPDE cells) (Fig. 8D, S6D, S7C, S7F) and in a few unknown cytoplasmic foci (Fig. 8E, arrow), but not in P bodies (0% in both cell lines) (Fig. 8E, S6E, S7D, S7G) [40]. Therefore, the interaction between eIF3f and hnRNP K may take place in stress granules or in other cytoplasmic foci, but not in P bodies. The quantification of the signals is shown in Fig. S7.

**Figure 9:**
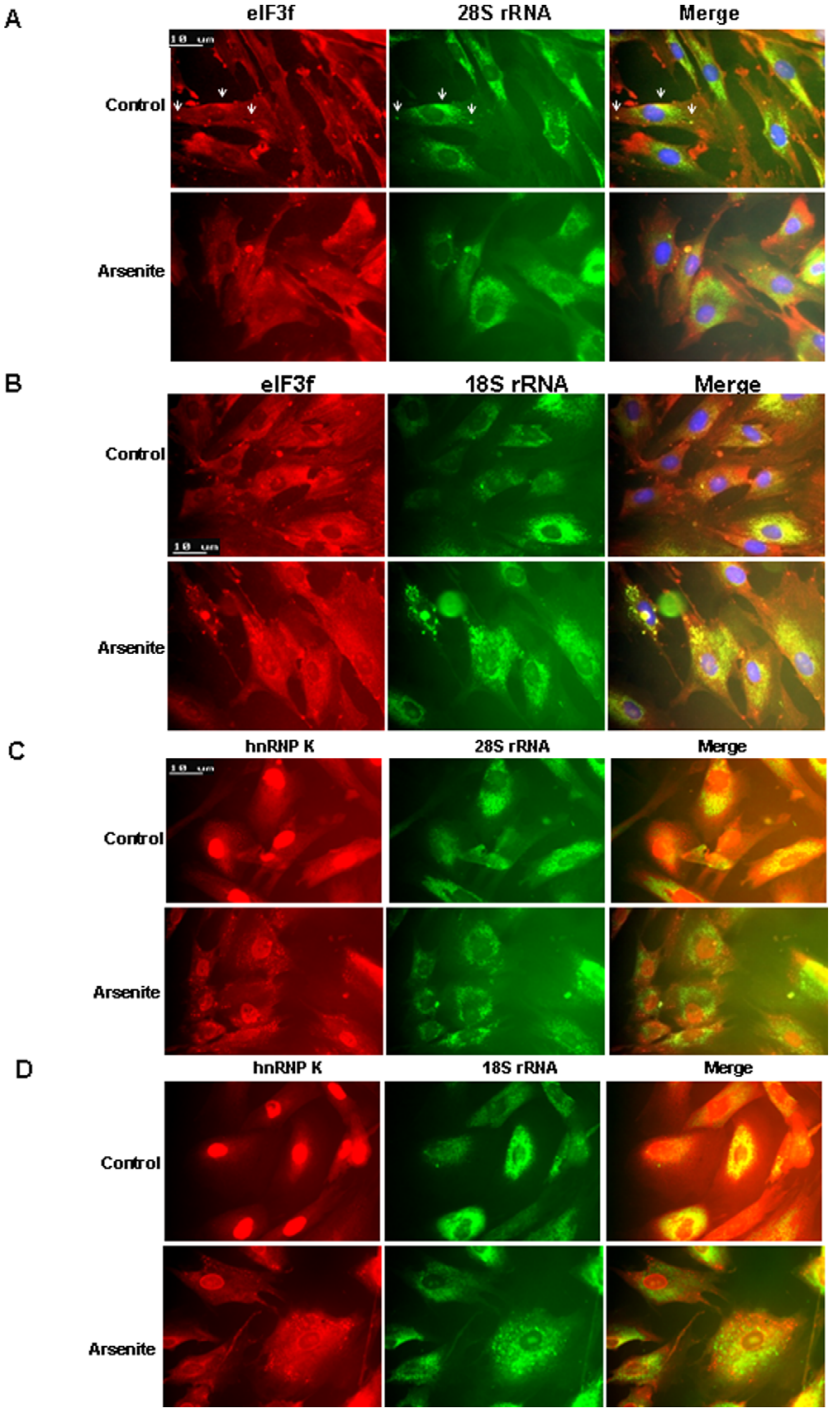
rRNA was accumulated in non-P body, non-stress granule cytoplasmic foci during stress. (A) eIF3f was partially co-localized with 28S rRNA in cytoplasmic foci. HFF-1 cells were treated with sodium arsenite for 45 minutes and labeled with 28S rRNA molecular beacon (FAM-tagged, green) followed by immunofluorescent analysis using eIF3f antibody (Texas Red-tagged secondary, red). (B) eIF3f was mostly co-localized with 18S rRNA in cytoplasmic foci in stressed cells. HFF-1 cells were treated with sodium arsenite for 45 minutes and labeled with 18S rRNA molecular beacon (FAM-tagged, green) followed by immunofluorescent analysis using eIF3f antibody (Texas Red-tagged secondary, red). (C) (D) hnRNP K was partially co-localized with rRNA in the perinuclear region in unstressed cells, but accumulated into cytoplasmic foci that were mostly not co-localized with 28S or 18S rRNA in stressed cells. HFF-1 cells were treated with sodium arsenite for 45 minutes and labeled with 28S or 18S rRNA molecular beacon (FAM-tagged, green) followed by immunofluorescent analysis using hnRNP K antibody (Texas Red-tagged secondary, red).

To investigate the association of eIF3f/hnRPN K with rRNAs, we combined immunofluorescent and molecular beacon assay [41]. In unstressed cells, we found that most of the 28S and 18S rRNA was perinuclear, with some rRNA in the nucleoli as expected (Fig. 9A, B). In the perinuclear region and in some cytoplasmic foci, eIF3f was partially co-localized with 18S and 28S rRNA (Fig. 9A, B, arrows). Under arsenite-induced stress, rRNA spread away from the nucleus, and 18S rRNA tended to accumulate into bigger cytoplasmic or perinuclear foci, but 28S rRNA was more diffused (Fig. 9A, B). As well, eIF3f also tended to accumulate into cytoplasmic foci where it was mostly co-localized with 18S, and partially with 28S rRNA (Fig. 9A, B), suggesting that the degradation mechanisms of 28S and 18S rRNA may be different, as shown in the nonfunctional rRNA degradation in yeast [37]. In unstressed cells, hnRNP K was partially co-localized with rRNA in the perinuclear region, but then accumulated into cytoplasmic foci (mostly stress granules) that were mostly not co-localized with rRNA in stressed cells (Fig. 9C, D). These results were consistent with our observation that the decreased binding of hnRNP K to rRNA was associated with increased rRNA degradation in stressed cells (Fig. 7). We further showed that rRNA was not co-localized with P bodies, but may be partially co-localized with Dcp1a diffusely in the perinuclear region (Fig. S8). Thus, rRNA degradation appears to occur in non-P body, non-stress granule cytoplasmic foci. eIF3f may play an important role in rRNA degradation by undermining the protective function of hnRNP K to rRNA.

## Discussion

We previously described decreased expression of eIF3f in pancreatic cancer [6], [9]. One of the molecular mechanisms of the decreased expression of eIF3f is loss of heterozygosity [9]. Other possible causes are mutation, epigenetic and transcription factor regulations (unpublished data by our group). We also previously showed that ectopic expression of eIF3f inhibited cell growth and induced apoptosis [6]. However whether decreased eIF3f can transform normal cells is unknown. In our current study, we further demonstrated that decreased eIF3f expression transformed a normal pancreatic epithelial cell line. Interestingly, decreased eIF3f expression also seemed to account for drug resistance in chemotherapy.

In the literature, some evidence suggests that alterations in eIF3 can contribute to tumorigenesis. Aberrant mRNA and protein levels of several eIF3 subunits have been detected in several different solid tumors and in several different cancer cell lines. Most eIF3 subunits (a, b, c, h, and i) are oncogenic. Interestingly, reduced expression and loss of heterozygosity (LOH) of eIF3e have been found in human breast cancer and lung cancer [42]. Clearly, deregulation of eIF3 subunits can contribute to tumorigenesis.

We previously showed that eIF3f is a negative regulator of translation [6]. Other investigators have observed a significant inhibition of overall protein synthesis in various cell types when cells are committed to apoptosis [43], [44], [45]. This downregulation of protein synthesis may either activate apoptosis or protect cells against noxious agents and ensure the conservation of resources needed for survival [46]. One of the 6 hallmarks of cancer is evading apoptosis [47]. Loss of eIF3f in pancreatic cancer may contribute to tumor cells' evading apoptosis via upregulation of protein synthesis. Another important mechanism for tumorigenesis is mitotic defect. As shown in our current study, misregulation of translation by eIF3f can cause aberrant cytokinesis and aneuploidy because of altered IRES-dependent translation. The mechanism by which eIF3f regulates protein synthesis is multileveled. One possible mechanism is direct binding and interference of the function of eIF3 translation initiation complex [17]. Another possible mechanism is further explored in the current paper – regulation of rRNA degradation and thus ribosome function. It is also very reasonable to suspect that eIF3f may directly or indirectly bind to mRNAs, since the eIF3 complex and some of the subunits of eIF3 is known to bind to mRNAs. Currently, whether the effect of eIF3f is universal and/or more specific for certain genes is not known. Studies are undergoing in our laboratory using RNA immunoprecipitation and microarray analysis to globally search for specific eIF3f-binding RNAs. The hope is to determine which coding and non-coding RNAs or consensus RNA sequence eIF3f binds to in order to find a potential pattern.

**Figure 10:**
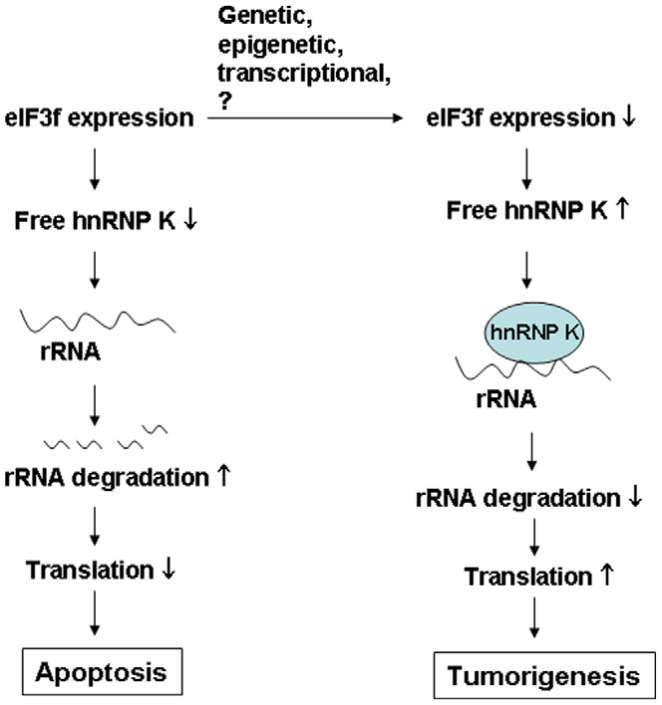
Diagram of our hypothesis. hnRNP K normally binds to and stabilizes rRNA. A physiologic expression level of eIF3f competes with rRNA for the binding of hnRNP K. This contributes to the maintenance of the homeostasis of rRNA level and translation in cells. Increased eIF3f expression contributes to apoptosis via hnRNP K sequestration and increased rRNA degradation. On the other hand during tumorigenesis, decreased expression of eIF3f releases more hnRNP K to bind to rRNA, which leads to increased rRNA stability, translation and cell growth.

One of the mechanisms of translation inhibition by eIF3f may be eIF3f-induced degradation of rRNA [6]. Exactly how eIF3f induces rRNA degradation is unclear. In our current study, we found evidence suggesting that eIF3f regulates rRNA degradation by interacting with hnRNP K, an RNA-binding protein. Our data support a model (Fig. 10) in which eIF3f sequesters hnRNP K to inhibit it's binding to rRNA, which leads to increased rRNA degradation and attenuated translation. In tumor cells, loss of eIF3f leads to increased binding of hnRNP K to rRNA, as well as imbalanced rRNA homeostasis and translation. The rRNA synthesis and degradation in a cell needs to be perfectly balanced in order to maintain homeostasis of protein synthesis. An increased rRNA level either through increased rRNA synthesis or through decreased rRNA degradation can be potentially oncogenic [11]. The regulatory mechanism of rRNA degradation in mammals is still not well understood; most studies have been carried out in yeast [37]. Our study demonstrated an unexpected function of a human translation initiation factor in rRNA degradation, linking translation initiation to the rRNA degradation mechanism and shedding light on the molecular mechanisms of the tumorigenic role of eIF3f in pancreatic cancer.

Currently, we still do not know where and how normal human rRNA is degraded during various physiologic or pathologic cellular processes, such as the cell cycle, stress, and apoptosis. In yeast, nonfunctional rRNA decay can be divided into 2 mechanistically distinct pathways [37]; mutated 18S rRNA can be seen in the P bodies, whereas mutated 25S rRNA (equivalent to 28S rRNA in humans) can be seen in the perinuclear foci in cytoplasm [37]. In the mRNA degradation mechanisms that have been primarily studied in the past, translational stalled mRNA accumulates in 2 types of cytoplasmic foci: P bodies and stress granules [38]. P bodies and stress granules are dynamic organizations of cytoplasmic ribonucleoproteins (RNPs) [38]. Translationally repressed mRNA, in conjunction with the mRNA decay machinery and other translation repressors, accumulates in P bodies. Stress granules typically contain poly(A)+ mRNA, 40S ribosomal subunits, eIF4E, eIF4G, eIF4A, eIF4B, poly(A)-binding protein (PABP), eIF3, and eIF2 [38]. In contrast to the widely held assumption, our data showed that eIF3f was localized both to P bodies and to stress granules. This observation suggests that eIF3f may play a unique role in mRNA degradation, besides its function in the translation initiation. Our data also suggest that normal rRNA decay mechanism may be different from nonfunctional rRNA decay. Under stress, normal rRNA was not localized either to P bodies or to stress granules. The exact components of the rRNA decay cytoplasmic foci need to be further investigated.

## Supporting Information

Figure S1 **Decreased eIF3f expression in pancreatic cancer and restoration of eIF3f expression in pancreatic cancer cells induced apoptosis.** (A) Hematoxylin and eosin (H&E) staining and Qdot immunohistochemistry (IHC) was performed on normal pancreas or pancreatic cancer tissue sections using eIF3f specific antibody, biotinylated secondary antibody and streptavidin-conjugated Qdot 655 (red). The nuclei were stained with DAPI (blue). The slides were evaluated by light and fluorescent microscopic examination and the representative images were taken. Note the loss of eIF3f protein (red) in pancreatic cancer cells. 400×. (B) Restoration of eIF3f expression induced apoptosis in pancreatic cancer cells. pcDNA3 or eIF3f transiently transfected BxPc3 and MiaPaCa-2 pancreatic cancer cells were analyzed for apoptosis by measuring caspase 3/7 activity.
(TIF)

Figure S2 **eIF3f-silencing in normal pancreatic epithelial cells led to malignant transformation.** (A) Immortalized normal human pancreatic ductal epithelial (HPDE) cells were transduced with 1 of the 5 predesigned MISSION eIF3f shRNA lentiviral particles (Sigma-Aldrich) individually, according to the manufacturer's instructions; 5 stable colonies were selected by puromycin resistance. Cells were harvested and total RNA extracted. 1 ug of RNA from each cell line were reverse-transcribed and relative eIF3f mRNA fold changes were examined by real time PCR and normalized to GAPDH mRNA as described in Materials and Methods. Cell lysates of the 5 cell lines were used in a Western blot analysis using eIF3f antibody. Vinculin was used as loading control. Densitometry analysis of the eIF3f bands normalized to corresponding vinculin is shown at the bottom. (B) In activated Kras^G12D^ HPDE cells, eIF3f-silenced cells had a higher proliferation rate. eIF3f stable knockdown (clone #5) and control HPDE cells were stably transfected with pcDNA3-Kras^G12D^. Same number of cells (5×10^4^ cells/plate) was seeded in triplicate on 100-mm plates and total cell numbers were counted every 2–3 days. (C) eIF3f-silenced cells had different cell morphology. Control and eIF3f RNAi HPDE cells were seeded in 6-well plate at about 50% confluent. Phase contrast images were taken using a digital camera attached to the microscope after 24 h. Note that the morphology of eIF3f-silenced HPDE cells mimics mesenchymal cells. (D) eIF3f-silenced cells had higher survival rate. eIF3f or control RNAi HPDE cells were treated with TNFα (0.1 µg/ml) to trigger apoptosis. Cell survival was measured at indicated times using MTT assay. Relative percentage of survival compared to control cells was shown.
(TIF)

Figure S3 **Generation of GFP-expressing HPDE cells in a 3D-cell culture.** (A) (B) eIF3f-silenced HPDE cells were stably transduced with a GFP lentivirus and positive cells were sorted by cell sorter. Representative phase contract and fluorescent images before and after sorting were shown in (A) and flow cytometry showed 98% of the cells are GFP positive after sorting (B). (C) These GFP-expressing green cells were used in an ex vivo 3D-culture system as described in Fig. 3. More representative confocal microscopy photos comparing control RNAi and eIF3f RNAi HPDE cells were shown here. Bar: 50 µm.
(TIF)

Figure S4 **eIF3f inhibited translation and decreased rRNAs in pancreatic cancer cells.** (A) Restoration of eIF3f expression decreased rRNAs. MiaPaCa-2 cells were transfected with pCMV-HA-eIF3f or pCMV-HA. Relative fold changes of eIF3f mRNA and rRNA levels were quantified by real time RT-PCR and normalized to G6PD mRNA. (B) (C) rRNA decrease caused by restoration of eIF3f is prior to peak apoptosis. MiaPaCa-2 cells were transfected with pcDNA3-eIF3f or pcDNA3. Cells were harvested at 4, 8 and 16 h after transfection and total RNA was isolated using RNeasy kit (QIAGEN). rRNAs (2.0 µg) were separated on an agarose gel and visualized by UV light (B). The 28S rRNA bands were quantified by densitometric analysis and shown at the bottom. Apoptosis was measured at the indicated time point after transfection using caspase Glo 3/7 kit (Promega) according to the manufacturer's instruction (C).
(TIF)

Figure S5 **eIF3f regulated rRNA stability through hnRNP K.** (A) Immunofluorescent staining of eIF3f (FITC, green) and hnRNP K (Cy3, red) in HFF-1 fibroblasts treated with staurosporine (10 ng/mL) for 24 h. (B) Ectopic expression of hnRNP K. HPDE cells were transfected with pcDNA4-hnRNP K or pcDNA4 control vector. Cells were treated with actinomycin D for 3 or 8 hours to block transcription 24 hours after transfection. Real time RT-PCR analysis was performed to quantify relative hnRNP K mRNA levels normalized to GAPDH mRNA. (C) hnRNP K expression was knocked down in MiaPaCa-2 cells by siRNA as in Fig. 7D. Actinomycin D was added to the cells 48 hours after transfection for 3 or 8 hours before harvest. Total RNA was isolated, DNase treated and real time RT-PCR analysis was performed to quantify relative hnRNP K mRNA fold change normalized to GAPDH mRNA. (D) HPDE cells were treated with etoposide (10 µM) for 24, 48 or 72 hours and cell survival was assessed by MTT assay. Average percentage change normalized to DMSO vehicle-treated cells from 3 independent experiments was shown.
(TIF)

Figure S6 **Localization of eIF3f/hnRNP K and their relationships with P body and stress granule.** (A) (B) HPDE and BxPC3 cells were treated with sodium arsenite (0.5 mM) for 45 minutes to trigger P body formation. Immunofluorescent study was performed using eIF3f and P body marker, Dcp1a, antibodies and FITC (green) or Texas Red (red) -tagged secondary antibodies as indicated. Arrowheads indicated that eIF3f is localized to non-P body cytoplasmic foci. (C) eIF3f inhibited P body formation. eIF3f RNAi and control RNAi HPDE cells were treated with sodium arsenite for 45 or 60 minutes and P body bearing and nonbearing cells were counted. A total of at least 200 cells were counted for each cell line. Percentage of P body bearing cells was calculated. Note that eIF3f-silenced cells had 2.5-fold increased natural occurring P body-bearing cells in untreated cells. (D)(E) hnRNP K was localized in stress granules, but not in P bodies. Immunofluorescent study was performed using hnRNP K (mouse), Rck (rabbit), or eIF4G (goat) antibodies and FITC-tagged anti-mouse (green), Texas Red-tagged anti-rabbit (red) or Cy3-tagged anti-goat (red) secondary antibodies in HPDE cells. Bar: 10 µm.
(TIF)

Figure S7 **Quantification of immunofluorescent signals.** The fluorescent dot signals (green, orange, red) in the cytoplasm were counted in the following samples. At least 100 dots were counted and percentages of each color signal were shown. (A)–(G) Quantification of fluorescent signals shown representatively in Fig. 8A and 8C, 8B, 8D, 8E, S6A–B, S6D, and S6E respectively.
(TIF)

Figure S8 **rRNA was not co-localized with P body.** HFF-1 cells were treated with sodium arsenite for 45 minutes and labeled with 28S or 18S rRNA molecular beacon (FAM-tagged, green) followed by immunofluorescent analysis using P body marker Dcp1a antibody (Texas Red-tagged secondary, red).
(TIF)
