# Correction: The Tumor Suppressive Role of eIF3f and Its Function in Translation Inhibition and rRNA Degradation

**DOI:** 10.1371/annotation/7c7feaa4-b9c5-4cd0-914b-b72499a16365

**Published:** 2012-05-31

**Authors:** Fushi Wen, Renyuan Zhou, Alex Shen, Andrew Choi, Diana Uribe, Jiaqi Shi

There was information missing in the Funding. The complete Funding information is: This work was supported by NCI/NIH grant CA133449, CA 158895, GI SPORE CA95060, and NIH Arizona Cancer Center Support Grant CA023074. The funders had no role in study design, data collection and analysis, decision to publish, or preparation of the manuscript.

