# Supplementary figures and images for: The Tumor Suppressive Role of eIF3f and Its Function in Translation Inhibition and rRNA Degradation
Source: PLoS One. 2012 Mar 23;7(3):e34194. doi: 10.1371/journal.pone.0034194 (PMC3311619; doi:10.1371/journal.pone.0034194)

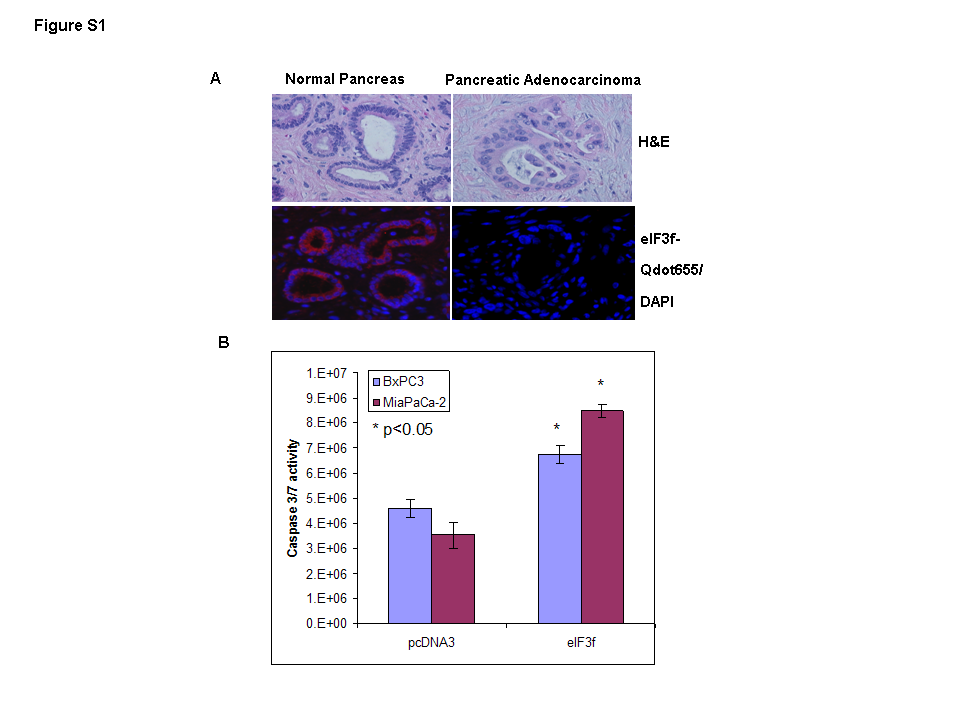

Supplement: Figure S1 — Decreased eIF3f expression in pancreatic cancer and restoration of eIF3f expression in pancreatic cancer cells induced apoptosis. (A) Hematoxylin and eosin (H&E) staining and Qdot immunohistochemistry (IHC) was performed on normal pancreas or pancreatic cancer tissue sections using eIF3f specific antibody, biotinylated secondary antibody and streptavidin-conjugated Qdot 655 (red). The nuclei were stained with DAPI (blue). The slides were evaluated by light and fluorescent microscopic examination and the representative images were taken. Note the loss of eIF3f protein (red) in pancreatic cancer cells. 400×. (B) Restoration of eIF3f expression induced apoptosis in pancreatic cancer cells. pcDNA3 or eIF3f transiently transfected BxPc3 and MiaPaCa-2 pancreatic cancer cells were analyzed for apoptosis by measuring caspase 3/7 activity. (TIF) [file pone.0034194.s001.tif]

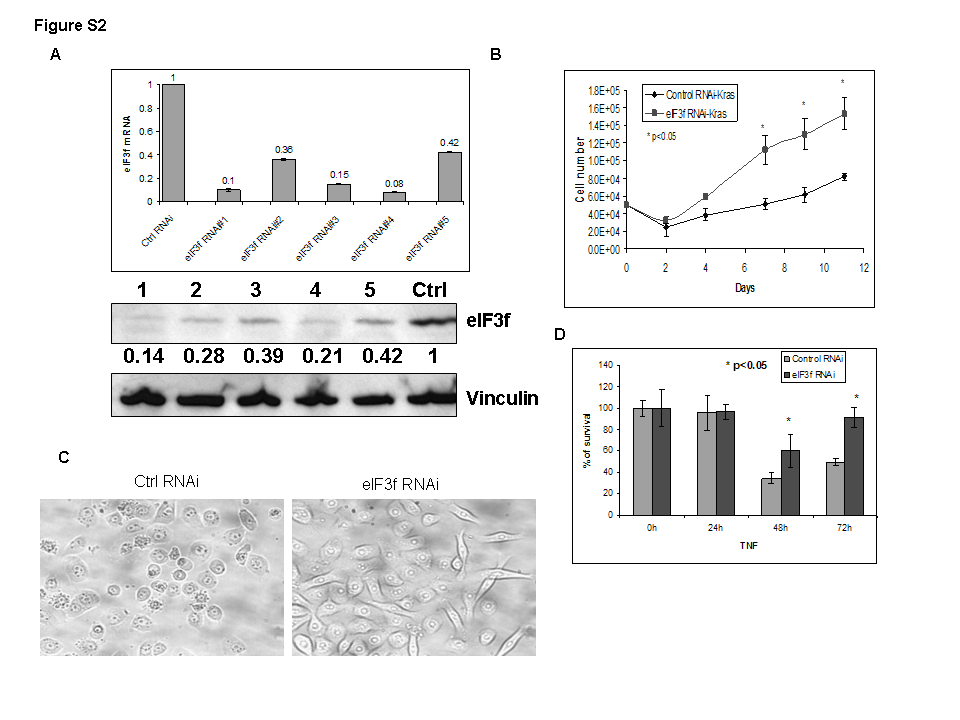

Supplement: Figure S2 — eIF3f-silencing in normal pancreatic epithelial cells led to malignant transformation. (A) Immortalized normal human pancreatic ductal epithelial (HPDE) cells were transduced with 1 of the 5 predesigned MISSION eIF3f shRNA lentiviral particles (Sigma-Aldrich) individually, according to the manufacturer's instructions; 5 stable colonies were selected by puromycin resistance. Cells were harvested and total RNA extracted. 1 ug of RNA from each cell line were reverse-transcribed and relative eIF3f mRNA fold changes were examined by real time PCR and normalized to GAPDH mRNA as described in Materials and Methods. Cell lysates of the 5 cell lines were used in a Western blot analysis using eIF3f antibody. Vinculin was used as loading control. Densitometry analysis of the eIF3f bands normalized to corresponding vinculin is shown at the bottom. (B) In activated KrasG12D HPDE cells, eIF3f-silenced cells had a higher proliferation rate. eIF3f stable knockdown (clone #5) and control HPDE cells were stably transfected with pcDNA3-KrasG12D. Same number of cells (5×10^4^ cells/plate) was seeded in triplicate on 100-mm plates and total cell numbers were counted every 2–3 days. (C) eIF3f-silenced cells had different cell morphology. Control and eIF3f RNAi HPDE cells were seeded in 6-well plate at about 50% confluent. Phase contrast images were taken using a digital camera attached to the microscope after 24 h. Note that the morphology of eIF3f-silenced HPDE cells mimics mesenchymal cells. (D) eIF3f-silenced cells had higher survival rate. eIF3f or control RNAi HPDE cells were treated with TNFα (0.1 µg/ml) to trigger apoptosis. Cell survival was measured at indicated times using MTT assay. Relative percentage of survival compared to control cells was shown. (TIF) [file pone.0034194.s002.tif]

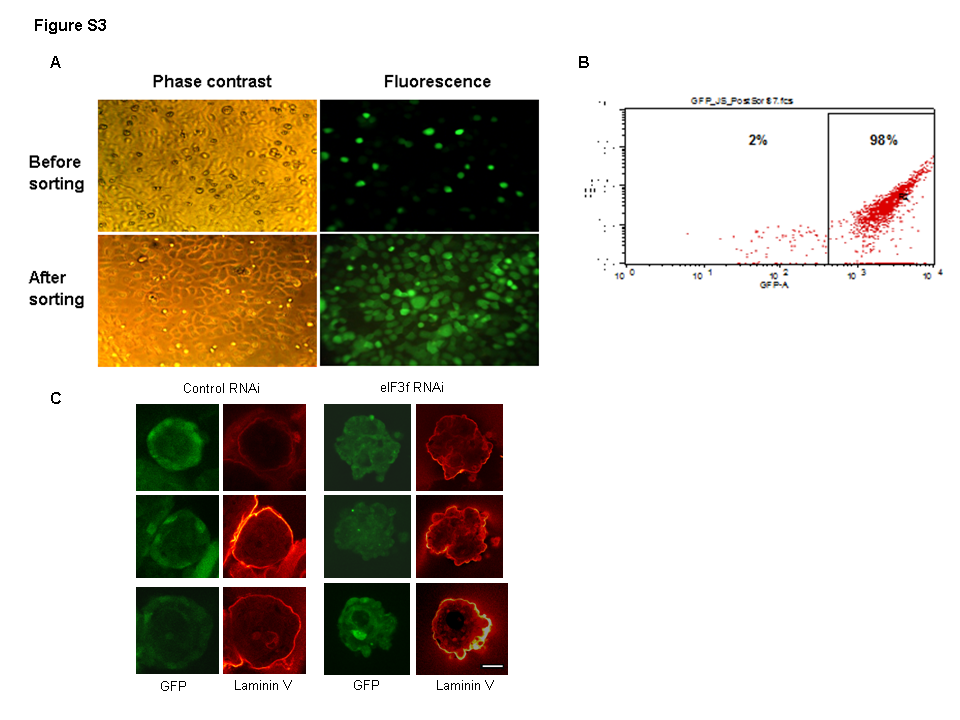

Supplement: Figure S3 — Generation of GFP-expressing HPDE cells in a 3D-cell culture. (A) (B) eIF3f-silenced HPDE cells were stably transduced with a GFP lentivirus and positive cells were sorted by cell sorter. Representative phase contract and fluorescent images before and after sorting were shown in (A) and flow cytometry showed 98% of the cells are GFP positive after sorting (B). (C) These GFP-expressing green cells were used in an ex vivo 3D-culture system as described in Fig. 3. More representative confocal microscopy photos comparing control RNAi and eIF3f RNAi HPDE cells were shown here. Bar: 50 µm. (TIF) [file pone.0034194.s003.tif]

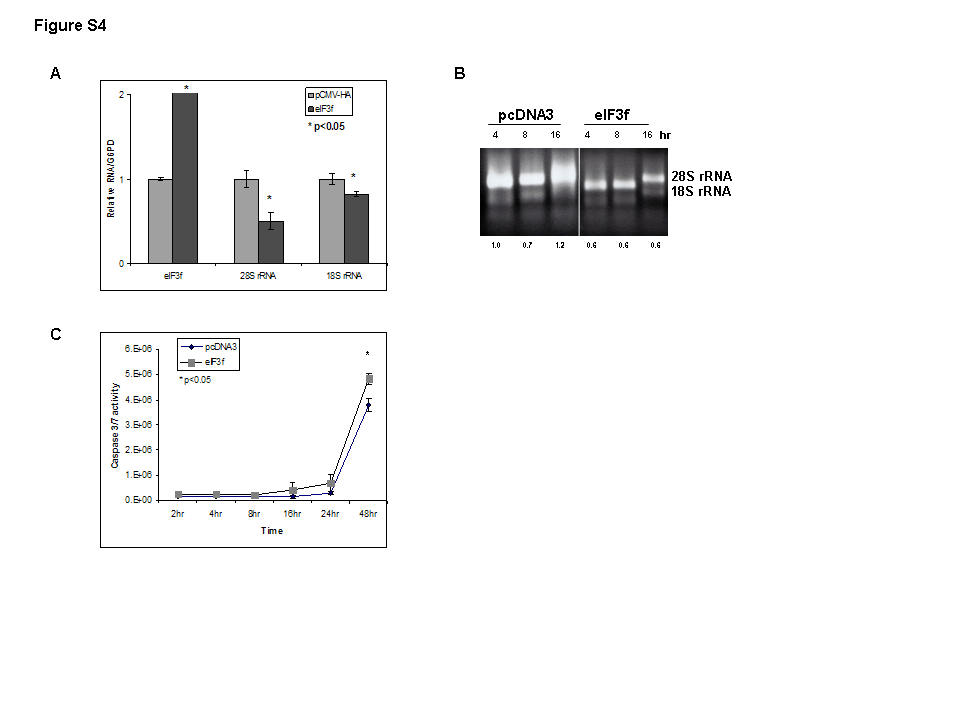

Supplement: Figure S4 — eIF3f inhibited translation and decreased rRNAs in pancreatic cancer cells. (A) Restoration of eIF3f expression decreased rRNAs. MiaPaCa-2 cells were transfected with pCMV-HA-eIF3f or pCMV-HA. Relative fold changes of eIF3f mRNA and rRNA levels were quantified by real time RT-PCR and normalized to G6PD mRNA. (B) (C) rRNA decrease caused by restoration of eIF3f is prior to peak apoptosis. MiaPaCa-2 cells were transfected with pcDNA3-eIF3f or pcDNA3. Cells were harvested at 4, 8 and 16 h after transfection and total RNA was isolated using RNeasy kit (QIAGEN). rRNAs (2.0 µg) were separated on an agarose gel and visualized by UV light (B). The 28S rRNA bands were quantified by densitometric analysis and shown at the bottom. Apoptosis was measured at the indicated time point after transfection using caspase Glo 3/7 kit (Promega) according to the manufacturer's instruction (C). (TIF) [file pone.0034194.s004.tif]

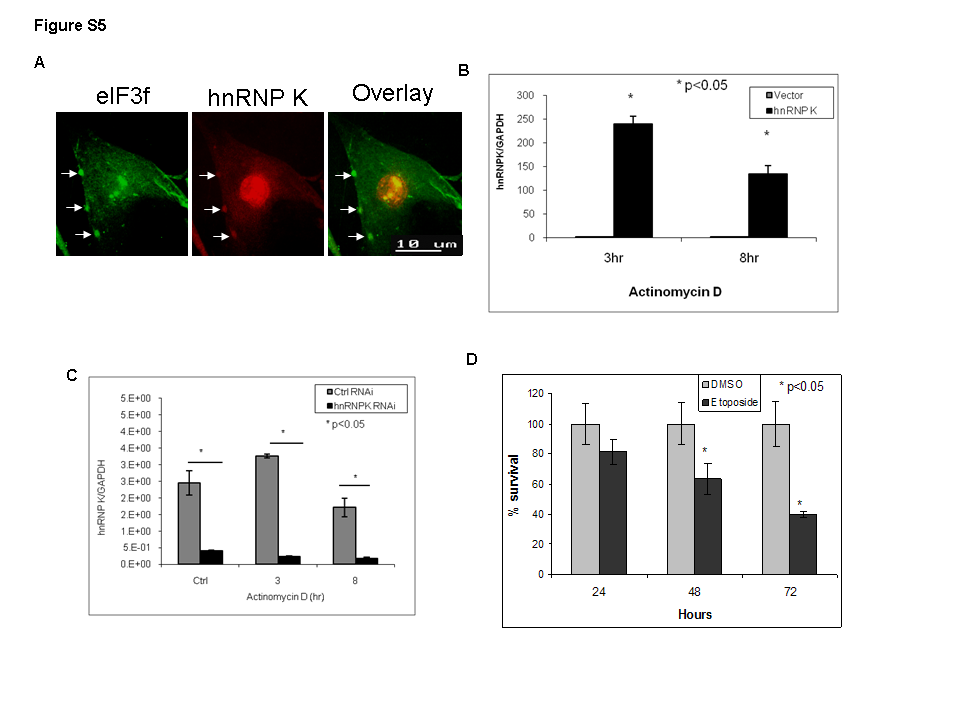

Supplement: Figure S5 — eIF3f regulated rRNA stability through hnRNP K. (A) Immunofluorescent staining of eIF3f (FITC, green) and hnRNP K (Cy3, red) in HFF-1 fibroblasts treated with staurosporine (10 ng/mL) for 24 h. (B) Ectopic expression of hnRNP K. HPDE cells were transfected with pcDNA4-hnRNP K or pcDNA4 control vector. Cells were treated with actinomycin D for 3 or 8 hours to block transcription 24 hours after transfection. Real time RT-PCR analysis was performed to quantify relative hnRNP K mRNA levels normalized to GAPDH mRNA. (C) hnRNP K expression was knocked down in MiaPaCa-2 cells by siRNA as in Fig. 7D. Actinomycin D was added to the cells 48 hours after transfection for 3 or 8 hours before harvest. Total RNA was isolated, DNase treated and real time RT-PCR analysis was performed to quantify relative hnRNP K mRNA fold change normalized to GAPDH mRNA. (D) HPDE cells were treated with etoposide (10 µM) for 24, 48 or 72 hours and cell survival was assessed by MTT assay. Average percentage change normalized to DMSO vehicle-treated cells from 3 independent experiments was shown. (TIF) [file pone.0034194.s005.tif]

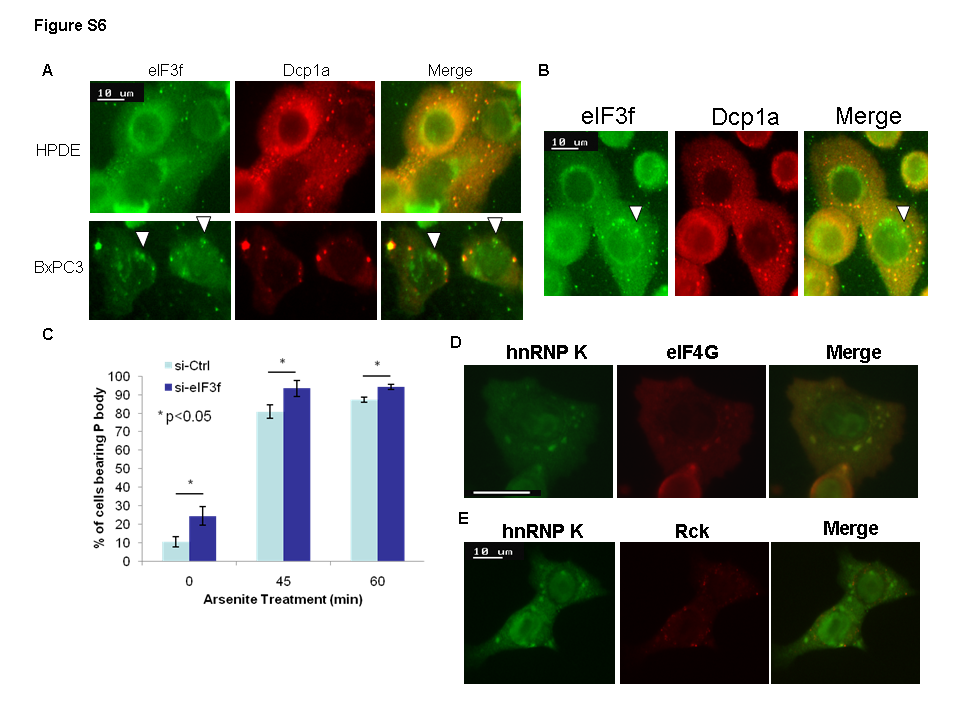

Supplement: Figure S6 — Localization of eIF3f/hnRNP K and their relationships with P body and stress granule. (A) (B) HPDE and BxPC3 cells were treated with sodium arsenite (0.5 mM) for 45 minutes to trigger P body formation. Immunofluorescent study was performed using eIF3f and P body marker, Dcp1a, antibodies and FITC (green) or Texas Red (red) -tagged secondary antibodies as indicated. Arrowheads indicated that eIF3f is localized to non-P body cytoplasmic foci. (C) eIF3f inhibited P body formation. eIF3f RNAi and control RNAi HPDE cells were treated with sodium arsenite for 45 or 60 minutes and P body bearing and nonbearing cells were counted. A total of at least 200 cells were counted for each cell line. Percentage of P body bearing cells was calculated. Note that eIF3f-silenced cells had 2.5-fold increased natural occurring P body-bearing cells in untreated cells. (D)(E) hnRNP K was localized in stress granules, but not in P bodies. Immunofluorescent study was performed using hnRNP K (mouse), Rck (rabbit), or eIF4G (goat) antibodies and FITC-tagged anti-mouse (green), Texas Red-tagged anti-rabbit (red) or Cy3-tagged anti-goat (red) secondary antibodies in HPDE cells. Bar: 10 µm. (TIF) [file pone.0034194.s006.tif]

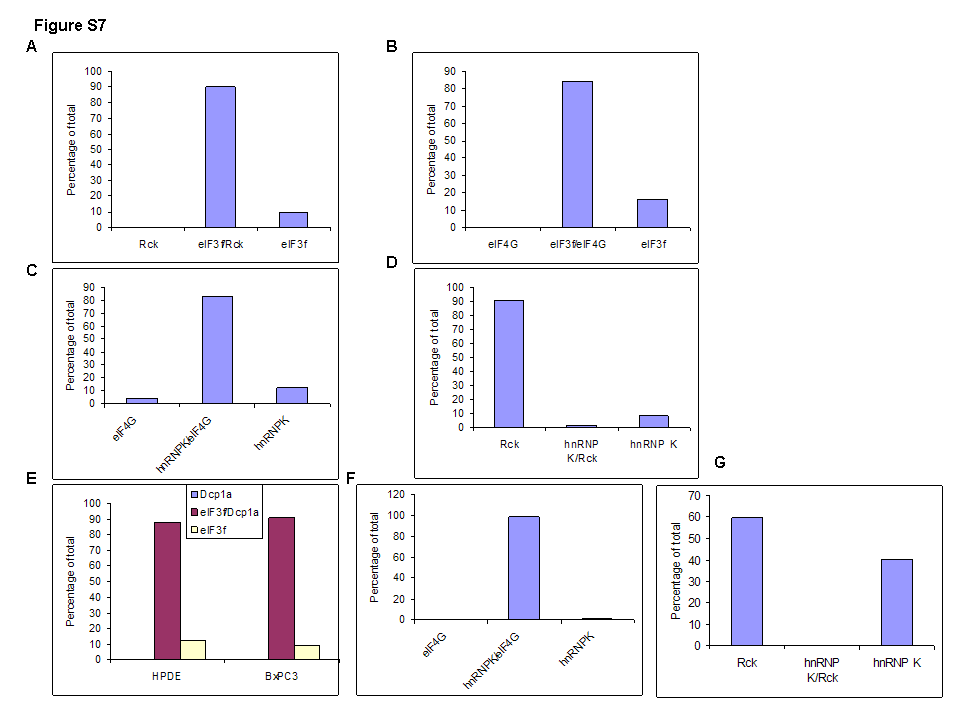

Supplement: Figure S7 — Quantification of immunofluorescent signals. The fluorescent dot signals (green, orange, red) in the cytoplasm were counted in the following samples. At least 100 dots were counted and percentages of each color signal were shown. (A)–(G) Quantification of fluorescent signals shown representatively in Fig. 8A and 8C, 8B, 8D, 8E, S6A–B, S6D, and S6E respectively. (TIF) [file pone.0034194.s007.tif]

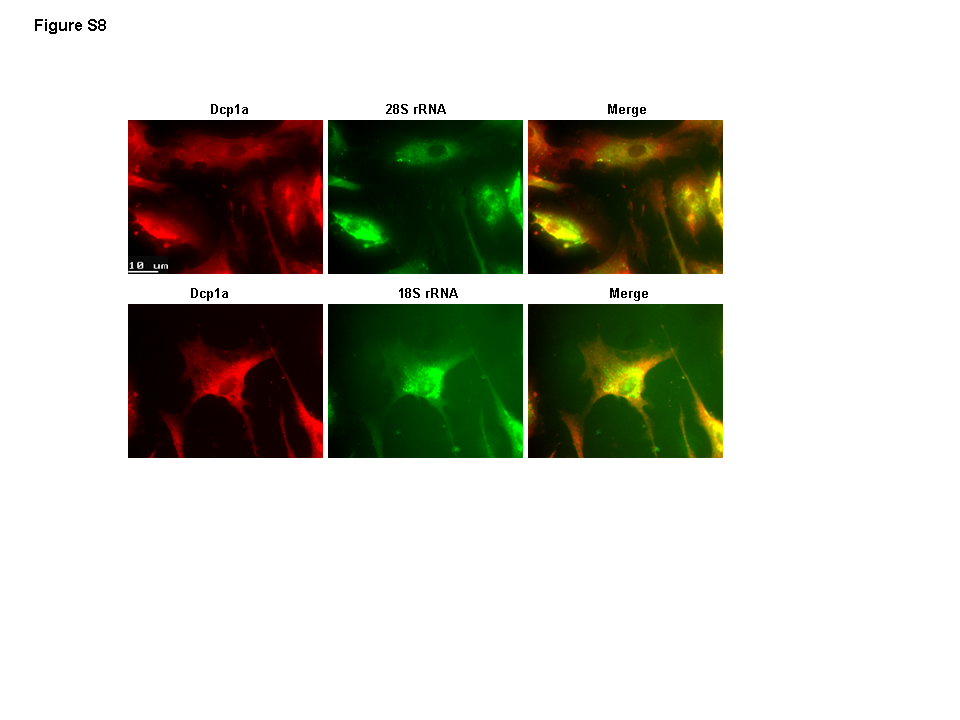

Supplement: Figure S8 — rRNA was not co-localized with P body. HFF-1 cells were treated with sodium arsenite for 45 minutes and labeled with 28S or 18S rRNA molecular beacon (FAM-tagged, green) followed by immunofluorescent analysis using P body marker Dcp1a antibody (Texas Red-tagged secondary, red). (TIF) [file pone.0034194.s008.tif]
